# Accelerated Diffusion-Based Sampling by the Non-Reversible Dynamics with Skew-Symmetric Matrices

**DOI:** 10.3390/e23080993

**Published:** 2021-07-30

**Authors:** Futoshi Futami, Tomoharu Iwata, Naonori Ueda, Issei Sato

**Affiliations:** 1 Communication Science Laboratories, NTT, Hikaridai, Seika-cho, “Keihanna Science City”, Kyoto 619-0237, Japan; tomoharu.iwata.gy@hco.ntt.co.jp (T.I.); naonori.ueda.fr@hco.ntt.co.jp (N.U.); 2 Department of Computer Science, Graduate School of Information Science and Technology, The University of Tokyo, 7-3-1 Hongo, Bunkyo-ku, Tokyo 113-0033, Japan; sato@g.ecc.u-tokyo.ac.jp

**Keywords:** Markov Chain Monte Carlo, Langevin dynamics, Hamilton Monte Carlo, non-reversible dynamics

## Abstract

Langevin dynamics (LD) has been extensively studied theoretically and practically as a basic sampling technique. Recently, the incorporation of non-reversible dynamics into LD is attracting attention because it accelerates the mixing speed of LD. Popular choices for non-reversible dynamics include underdamped Langevin dynamics (ULD), which uses second-order dynamics and perturbations with skew-symmetric matrices. Although ULD has been widely used in practice, the application of skew acceleration is limited although it is expected to show superior performance theoretically. Current work lacks a theoretical understanding of issues that are important to practitioners, including the selection criteria for skew-symmetric matrices, quantitative evaluations of acceleration, and the large memory cost of storing skew matrices. In this study, we theoretically and numerically clarify these problems by analyzing acceleration focusing on how the skew-symmetric matrix perturbs the Hessian matrix of potential functions. We also present a practical algorithm that accelerates the standard LD and ULD, which uses novel memory-efficient skew-symmetric matrices under parallel-chain Monte Carlo settings.

## 1. Introduction

Sampling is one of the most widely used techniques for the approximation of posterior distribution in Bayesian inference [1]. Markov Chain Monte Carlo (MCMC) is widely used to obtain samples. In MCMC, Langevin dynamics (LD) is a popular choice for sampling from high-dimensional distributions. Each sample in LD moves toward a gradient direction with added Gaussian noise. LD efficiently explore around a mode of a target distribution using the gradient information without being trapped by local minima thanks to added Gaussian noise. Many previous studies theoretically and numerically proved LD’s superior performance [2,3,4,5]. Since non-reversible dynamics generally improves mixing performance [6,7], research on introducing non-reversible dynamics to LD for better sampling performance is attracting attention [8].

There are two widely known non-reversible dynamics for LD. One is underdamped Langevin dynamics (ULD) [9], which uses second-order dynamics. The other introduces perturbation, which consists of multiplying the skew-symmetric matrix by a gradient [8]. Here, we refer to the matrix as skew matrices for simplicity and this perturbation technique as skew acceleration. Much research has been done on ULD theoretically [9,10,11] and ULD is widely used in practice, which is also known as stochastic gradient Hamilton Monte Carlo [12]. In contrast, the application of the skew acceleration for standard Bayesian models is quite limited even though it is expected to show superior performance theoretically [8].

For example, skew acceleration has been analyzed focusing on sampling from Gaussian distributions [13,14,15,16,17], although assuming Gaussian distributions in Bayesian models is restrictive in practice. A recent study [8] theoretically showed that skew acceleration accelerates the dynamics around the local minima and saddle points for non-convex functions. Another work [18] clarified that the skew acceleration theoretically and numerically improves mixing speed when used as interactions between chains in parallel sampling schemes for non-convex Bayesian models.

Compared to ULD, what seems to be lacking for skew acceleration is a theoretical understanding of issues that are important to practitioners. The most significant problem is that no theory exists for selecting skew matrices. In existing studies, introducing a skew matrix into LD results in equal or faster convergence, denoting that a bad choice of skew matrix results in no acceleration. Thus, choosing appropriate skew matrices is critical. Furthermore, although ULD’s acceleration has been analyzed quantitatively, existing studies have only analyzed skew acceleration qualitatively. Thus, it is difficult to justify the usefulness of skew acceleration in practice compared to ULD. Another issue is that introducing skew matrices requires a vast memory cost in many practical Bayesian models.

The purpose of this study is to solve these problems from theoretical and numerical viewpoints and establish a practical algorithm for skew acceleration. The following are the two major contributions of this work.

Our contribution 1: We present a convergence analysis of skew acceleration for standard Bayesian model settings, including non-convex potential functions using Poincaré constants [19]. The major advantage of Poincaré constants is that we can analyze skew acceleration through a Hessian matrix and its eigenvalues and develop a practical theory about the selection of *J* and the quantitative assessment of skew acceleration.

Furthermore, we propose skew acceleration for ULD and present convergence analysis for the first time. Since ULD shows faster convergence than LD, combining skew acceleration with ULD is promising.

Our contribution 2: We develop a practical skew accelerated sampling algorithm for a parallel sampling setting with novel memory-efficient skew matrices. Since a naive implementation of skew acceleration requires a large memory cost to store skew matrices, memory-efficiency is critical in practice. We also present a non-asymptotic theoretical analysis for our algorithm in both LD and ULD settings under a stochastic gradient and Euler discretization. We clarify that introducing skew matrices accelerates the convergence of continuous dynamics, although it increases the discretization and stochastic gradient error. Then to the best of our knowledge, we propose the first algorithm that adaptively controls this trade-off using the empirical distribution of the parallel sampling scheme.

Finally, we verify our algorithm and theory in practical Bayesian problems and compare it with other sampling methods.

Notations: $I_{d}$ denotes a d×d identity matrix. Capital letters such as *X* represent random variables, and lowercase letters such as *x* represent non-random real values. ·, ∥·∥ and |·| denote Euclidean inner products, distances and absolute values.

## 2. Preliminaries

In this section, we briefly introduce the basic settings of LD and non-reversible dynamics for the posterior distribution sampling in Bayesian inference.

### 2.1. LD and Stochastic Gradient LD

First, we introduce the notations and the basic settings of LD and stochastic gradient LD (SGLD), which is a practical extension of LD. Here $z_{i}$ denotes a data point in space Z, |Z| denotes the total number of data points, and $x\in R^{d}$ corresponds to the parameters of a given model, which we want to sample. Our goal is to sample from the target distribution with density $d\pi \left(x\right)\propto e^{-\beta U(x)}dx$, where potential function U(x) is the summation of $u:R^{d}\times Z\to R$, i.e., $U\left(x\right)=\frac{1}{\left|Z\right|}\sum_{i=1}^{\left|Z\right|}u\left(x,z_{i}\right)$. Function u(·,·) is continuous and non-convex. The explicit assumptions made for it are discussed in Section 3.1. The SGLD algorithm [2,3] is given as a recursion:

(1) $$\begin{array}{c} X_{k+1}=X_{k}-h\nabla \hat{U}\left(X_{k}\right)+\sqrt{2h\beta^{-1}}\epsilon_{k}, \end{array}$$

where $h\in R^{+}$ is a step size, $\epsilon_{k}\in R^{d}$ is a standard Gaussian random vector, β is a temperature parameter of π, and $\nabla \hat{U}\left(X_{k}\right)$ is a conditionally unbiased estimator of true gradient $\nabla U(X_{k})$. This unbiased estimate of the true gradient is suitable for large-scale data set since we can use not the full gradient, but a stochastic version obtained through a randomly chosen subset of data at each time step. This means that we can reduce the computational cost to calculate the gradient at each time step.

The discrete time Markov process in Equation (1) is the discretization of the continuous-time LD [2]:

(2) $$\begin{array}{c} dX_{t}=-\nabla U\left(X_{t}\right)dt+\sqrt{2\beta^{-1}}dw_{t}, \end{array}$$

where $w_{t}$ denotes the standard Brownian motion in $R^{d}$. The stationary measure of Equation (2) is $d\pi \left(x\right)\propto e^{-\beta U(x)}dx$.

### 2.2. Poincaré Inequality and Convergence Speed

In sampling, we are interested in the convergence speed to the stationary measure. The speed is often characterized by the *the generator* associated with Equation (2) and defined as:

(3) $$\begin{array}{cc} Lf(X_{t}): & =\lim_{s\to 0^{+}}\frac{E\left(f\left(X_{t+s}\right)|X_{t}\right)-f\left(X_{t}\right)}{s}\\ \; & =\left(-\nabla U\left(X_{t}\right)\cdot \nabla +\beta^{-1}\Delta \right)f\left(X_{t}\right), \end{array}$$

where Δ denotes a standard Laplacian on $R^{d}$ and f∈D(L) and $D\left(L\right)\subset L^{2}\left(\pi \right)$ denote the L domain. This −L is a self-adjoint operator, which has only discrete spectrums (eigenvalues). π with L has a *spectral gap* if the smallest eigenvalue of −L (other than 0) is positive. We refer to it as $\rho_{0}\left(>0\right)$. This spectral gap is closely related to Poincaré inequality. Internal energy is defined:

(4) $$\begin{array}{c} E\left(f\right):=-\int_{R^{d}}fLfd\pi . \end{array}$$

Please note that E(f)>0 is satisfied. Then π with L satisfies the Poincaré inequality with constant *c*, if for any f∈D(L), π with L satisfies:

(5) $$\begin{array}{c} \int f^{2}d\pi -{\left(\int fd\pi \right)}^{2}\leq cE\left(f\right). \end{array}$$

The spectral gap characterizes this constant $c\leq \frac{1}{\rho_{0}}$, which holds (see Appendix A.2 for details). We refer to best constant *c* as the Poincaré constant [19]. For notational simplicity, we define $m_{0}:=\frac{1}{c}$ and refer to this $m_{0}$ as the Poincaré constant.

In sampling, crucially, Poincaré inequality dominates the convergence speed in $\chi^{2}$ divergence:

(6) $$\begin{array}{c} \int {\left(\frac{d\mu_{t}}{d\pi }-1\right)}^{2}d\pi :=\chi^{2}\left(\mu_{t}∥\pi \right)\leq e^{-\frac{2m_{0}}{\beta }t}\chi^{2}\left(\mu_{0}∥\pi \right), \end{array}$$

where $\mu_{t}$ denotes the measure at time *t* induced by Equation (2) and $\mu_{0}$ is the initial measure (see Appendix A.3 for details). Thus, the larger Poincaré constant $m_{0}$ is, the faster convergence we have.

### 2.3. Non-Reversible Dynamics

In this section, we introduce the non-reversible dynamics. π with L is reversible if for any test function f,g∈D(L), π with L satisfies

(7) $$\begin{array}{c} \int_{R^{d}}fLgd\pi =\int_{R^{d}}gLfd\pi . \end{array}$$

If this is not satisfied, π with L is non-reversible [19].

We introduce two non-reversible dynamics for LD. The first is ULD, which is given as

(8) $$\begin{array}{c} \begin{array}{cc} \; & dX_{t}=\Sigma^{-1}V_{t}dt,\\ \; & dV_{t}=-\nabla U\left(X_{t}\right)dt-\gamma \Sigma^{-1}V_{t}dt+\sqrt{2\gamma \beta^{-1}}dw_{t}, \end{array} \end{array}$$

where $V\in R^{d}$ is an auxiliary random variable, γ∈R is a positive constant, and Σ is the variance of the stationary distribution of auxiliary random variable *V*. The stationary distribution is $\tilde{\pi }:=\pi ⊗N\left(0,\Sigma \right)\propto e^{-\beta U\left(x\right)-\frac{1}{2}\Sigma^{-1}{∥v∥}^{2}}$, where N denotes a Gaussian distribution. The superior performance of ULD compared with LD has been studied rigorously [9,10,11]. ULD’s convergence speed is also characterized by the Poincaré constant [20]. In practice, we use discretization and the stochastic gradient for ULD, which is called the stochastic gradient Hamilton Monte Carlo (SGHMC) [10]. The second non-reversible dynamics is the skew acceleration given as

(9) $$\begin{array}{c} dX_{t}=-\left(I+\alpha J\right)\nabla U\left(X_{t}\right)dt+\sqrt{2\beta^{-1}}dw_{t}, \end{array}$$

where *J* is a real value skew matrix and $\alpha \in R^{+}$ is a positive constant. We call this dynamics S-LD. The stationary distribution of S-LD is still π, and S-LD shows faster convergence and smaller asymptotic variance [13,14,15,18].

## 3. Theoretical Analysis of Skew Acceleration

In this section, we present a theoretical analysis of skew acceleration in LD and ULD in standard Bayesian settings. We analyze acceleration through the Poincaré constant and connect it with the eigenvalues of the Hessian matrix, which allows us to obtain a practical criterion to choose skew matrices and quantitatively evaluate acceleration. We focus on a setting where a continuous SDE and a full gradient of the potential function is used in this section. The discretized SDE and stochastic gradient are discussed in Section 4.

### 3.1. Acceleration Characterization by the Poincaré Constant

First, we introduce the same four assumptions as a previous work [2], which showed the existence of the Poincaré constant about $m_{0}$ for LD (see Appendix C for details).

**Assumption 1.**  *(Upper bound of the potential function at the origin) Function u takes nonnegative real values and is twice continuously differentiable on $R^{d}$, and constants A and B exist such that for all z∈Z,*

(10) $$\begin{array}{c} |u(0,z)|\leq A,\;∥\nabla u(0,z)∥\leq B. \end{array}$$

**Assumption 2.**  *(Smoothness) Function u has Lipschitz continuous gradients; for all z∈Z, positive constant M exists for all $x,y\in R^{d}$,*

(11) $$\begin{array}{c} ∥\nabla u(x,z)-\nabla u(y,z)∥\leq M∥x-y∥. \end{array}$$

**Assumption 3.**  *(Dissipative condition) Function u satisfies the (m,b)-dissipative condition for all z∈Z; for all $x\in R^{d}$, m>0 and b≥0 exist such that*

(12) $$\begin{array}{c} -x\cdot \nabla u\left(x,z\right)\leq -{m∥x∥}^{2}+b. \end{array}$$

**Assumption 4.**  *(Initial condition) Initial probability distribution $\mu_{0}$ of $X_{0}$ has a bounded and strictly positive density $p_{0}$, and for all $x\in R^{d}$,*

(13) $$\begin{array}{c} \kappa_{0}:=\log\int_{R^{d}}e^{{∥x∥}^{2}}p_{0}\left(x\right)dx<\infty . \end{array}$$

Please note that these assumptions allow us to consider the non-convex potential functions, which are common in practical Bayesian models. Furthermore, we make the following assumption about *J*.

**Assumption** **5.**  *The operator norm of J is bounded:*

(14) $$\begin{array}{c} {∥J∥}_{2}\leq 1. \end{array}$$

*This means that the largest singular value of J is below 1.*

Under these assumptions, we present the convergence behavior of skew acceleration using the Poincaré constant. First, we present the following S-LD result.

**Theorem** **1.** *Under Assumptions 1–5, the S-LD of Equation (9) has exponential convergence,*

(15) $$\begin{array}{c} \chi^{2}\left(\mu_{t}^{\alpha }∥\pi \right)\leq e^{-\frac{2m(\alpha )}{\beta }t}\chi^{2}\left(\mu_{0}∥\pi \right), \end{array}$$

*where $\mu_{t}^{\alpha }$ is the measure at time t induced by S-LD and m(α) is the Poincaré constant of S-LD defined by its generator*

(16) $$\begin{array}{c} L_{\alpha }f\left(x\right):=\left(-\left(I+\alpha J\right)\nabla U\left(x\right)\cdot \nabla +\beta^{-1}\Delta \right)f\left(x\right). \end{array}$$

*Furthermore, m(α) satisfies $m\left(\alpha \right)\geq m_{0}$.*

The proof is shown in Appendix C. This theorem states that introducing the skew matrices accelerates the convergence of LD by improving the convergence rate from $m_{0}$ to m(α). Although [18] obtained a similar result, we used the Poincaré constant and derived an explicit criterion when $m\left(\alpha \right)=m_{0}$ holds, as we discuss below.

Next, we also introduce skew acceleration in ULD. Since ULD shows faster convergence than LD in standard Bayesian settings [10,11], it is promising to combine skew acceleration with ULD to obtain a more efficient sampling algorithm. For that purpose, we propose the following SDE:

(17) $$\begin{array}{cc} \; & dX_{t}=\Sigma^{-1}V_{t}dt+\alpha_{1}J_{1}\nabla U\left(X_{t}\right)dt, \end{array}$$

(18) $$\begin{array}{cc} \; & dV_{t}=-\nabla U\left(X_{t}\right)dt-\gamma \left(\Sigma^{-1}+\alpha_{2}J_{2}\right)V_{t}dt+\sqrt{2\gamma \beta^{-1}}dw_{t}, \end{array}$$

where $J_{1}$ and $J_{2}$ are real value skew matrices and $\alpha_{1}$ and $\alpha_{2}$ are positive constants. We assume that $J_{1}$ and $J_{2}$ satisfy Assumption 5. We refer to this method as skew underdamped Langevin dynamics (S-ULD) whose stationary distribution is $\tilde{\pi }=\pi ⊗N\left(0,\Sigma \right)\propto e^{-\beta U\left(x\right)-\frac{1}{2}\Sigma^{-1}{∥v∥}^{2}}$. See Appendix B for details, which include discussions on other combinations of skew matrices. As for S-ULD, we need an additional assumption about the initial condition of $V_{0}$:

**Assumption 6.**  *(Initial condition) Initial probability distribution $\mu_{0}\left(x,v\right)$ of $\left(X_{0},V_{0}\right)$ has a bounded and strictly positive density $p_{0}$ that satisfies,*

(19) $$\begin{array}{c} \kappa_{0}:=\log\int_{R^{2d}}e^{{∥x∥}^{2}+{∥v∥}^{2}}p_{0}\left(\left(x,v\right)\right)dxdv<\infty . \end{array}$$

We then provide the following convergence theorem that resembles S-LD.

**Theorem** **2.** *Under Assumptions 1–3, 5, 6, S-ULD has exponential convergence in $\chi^{2}$ divergence and its convergence rate is also characterized by m(α) as defined in Theorem 1. S-ULD’s convergence equals or exceeds ULD, of which convergence rate is characterized by $m_{0}$.*

See Appendix C.2 for details. From these theorems, we confirmed that skew acceleration is effective in both S-LD and S-ULD, and the convergence speed is characterized by Poincaré constant m(α) defined by Equation (16).

### 3.2. Skew Acceleration from the Hessian Matrix

Our goal is to clarify what choices of *J* induce $m\left(\alpha \right)>m_{0}$, which leads to acceleration. Therefore, we discuss how Poincaré constant m(α) is connected to the eigenvalues and eigenvectors of the perturbed Hessian matrix $\left(I+\alpha J\right)\nabla^{2}U\left(x\right)$. Next, we introduce the notations. We express the Hessian of U(x) as H(x) and the perturbed Hessian matrix as $H^{\prime }\left(x\right):=\left(I+\alpha J\right)H\left(x\right)$. Please note that *H* is a real symmetric matrix, which has real eigenvalues and diagonalizable. On the other hand, since $H^{\prime }$ is not symmetric, it has complex eigenvalues, although diagonalization is not assured (see Appendix E). We express pairs of eigenvectors and eigenvalues of $H^{\prime }\left(x\right)$ as ${\left\{\left(v_{i}^{\alpha }\left(x\right),\lambda_{i}^{\alpha }\left(x\right)\right)\right\}}_{i=1}^{d}$, which are ordered as Re(λ1α(x)))≤⋯≤Re(λdα(x)). Here, Re(λ1α(x)) expresses the real part of complex value $\lambda_{1}^{\alpha }$ and Im denotes the imaginary part. We express those of H(x) as ${\left\{\left(v_{i}^{0}\left(x\right),\lambda_{i}^{0}\left(x\right)\right)\right\}}_{i=1}^{d}$ and order them as $\lambda_{1}^{0}\left(x\right)\leq \cdots \leq \lambda_{d}^{0}\left(x\right)$.

#### 3.2.1. Strongly Convex Potential Function

Assume that *U* is an m-strongly convex function, where for all $x\in R^{d}$, $m\leq \lambda_{1}^{0}\left(x\right)$ holds. Poincaré constant $m_{0}$ of LD satisfies $m_{0}=m$ [19]. For the skew acceleration, since Poincaré constant satisfies $m\left(\alpha \right)=m^{\prime }\left(\alpha \right)$, where $m^{\prime }\left(\alpha \right)$ is the best constant that satisfies, for all *x*, m′(α)≤Reλ1α(x) (see Appendix D.1). Therefore, studying the Poincaré constant is equivalent to studying the smallest (real part of the) eigenvalue of the Hessian matrix. Thus, the relation between $\lambda_{1}^{0}\left(x\right)$ and Reλ1α(x) must be studied. The following theorem describes how the skew matrices change the smallest eigenvalue.

**Theorem** **3.** *For all $x\in R^{d}$, the real parts of the eigenvalues of $H^{\prime }$ satisfy*

(20) m≤λ10(x)≤Reλ1α(x)≤⋯≤Reλdα(x)≤λd0(x).

*The condition of λ10(x)=Reλ1αx)) is shown in Remark 1.*

**Remark** **1.** *Denote the set of the eigenvectors of eigenvalue $\lambda_{1}^{0}\left(x\right)$ as $V_{1}^{0}$. If $V_{1}^{0}=\left\{v\right\}$ and Jv=0, then λ10(x)=Reλ1αx)) holds. If the cardinality of set $V_{1}^{0}$ is larger than* 1, *and vectors $v,v^{\prime }\in V_{1}^{0}$ exist, such that λ10αJv=(Imλ1α)v′ and λ10αJv′=−(Imλ1α)v, then λ10(x)=Reλ1αx)) holds.*

Refer to Appendix F for the proof. This is an extension of previous work [8,13]. If λ10(x)<Reλ1α(x) is satisfied for all *x*, we have $m_{0}<m\left(\alpha \right)$, i.e., acceleration occurs. We discuss how to construct *J* such that λ10(x)<Reλ1α(x) holds in Section 3.3.

#### 3.2.2. Non-Convex Potential Function

**Figure 1:**
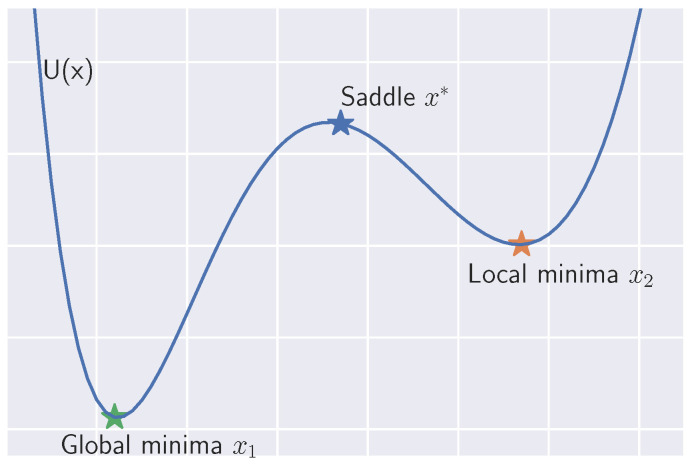
Double-potential example: Poincaré constant is related to the eigenvalue at $x^{*}$.

The previous work [21] clarified that the Poincaré constant of the non-convex function is characterized by the negative eigenvalue of the saddle point. As shown in Figure 1, denote $x_{1}$ as the global minima, and $x_{2}$ is the local minima which has the second smallest value in U(x). We express the saddle point with index one, i.e., there is only one negative eigenvalue at the point, between $x_{1}$ and $x_{2}$ as $x^{*}$. This means that the eigenvalues of $H(x^{*})$ satisfies $\lambda_{1}^{0}\left(x^{*}\right)<0<\lambda_{2}^{0}\left(x^{*}\right)<\cdots <\lambda_{d}^{0}\left(x^{*}\right)$. Ref. [21] clarified that the saddle point $x^{*}$ characterizes the Poincaré constant as

(21) $$\begin{array}{c} m_{0}^{-1}\propto \frac{1}{|\lambda_{1}\left(x^{*}\right)|}e^{\beta (U\left(x^{*}\right)-U\left(x_{1}\right)-U\left(x_{2}\right))}. \end{array}$$

When skew matrices are introduced, [8] clarified the following relation:

**Theorem 4.**  *([8]) $\lambda_{1}^{\alpha }\left(x^{*}\right)\leq \lambda_{1}^{0}\left(x^{*}\right)<0$ and equality holds only if $Jv_{1}^{\alpha }\left(x^{*}\right)=0$.*

Note $\lambda_{1}^{\alpha }\left(x^{*}\right)$ is not a complex number. Thus, the skew acceleration reduces the negative eigenvalue and leads to a larger Poincaré constant (see Appendix D.2) and results in faster convergence.

In conclusion, introducing the skew matrix changes the Hessian’s eigenvalues and increase the Poincaré constant. If λ10(x)≠Reλ1α(x) is satisfied, this leads to faster convergence for both convex and non-convex potential functions.

### 3.3. Choosing J

In this section, we present a method for choosing *J* that leads to λ10(x)≠Reλ1α(x) to ensure the acceleration based on the equality conditions in Theorems 3 and 4. Combining these theorems, we obtain the following criterion:

**Remark** **2.** *Given a point x, λ10(x)≠Reλ1α(x) holds if either the following conditions are satisfied: (i) when $V_{1}^{0}=\left\{v\right\}$, Jv≠0 is satisfied. (ii) when $|V_{1}^{0}|>1$, Jv≠0 holds for any $v\in V_{1}^{0}$, and for any $v,v^{\prime }\in V_{1}^{0}$, λ10αJv=(Imλ1α)v′ and λ10αJv′=−(Imλ1α)v are not satisfied.*

The first condition (i) is easily satisfied if we choose *J* such that KerJ={0}. On the other hand, the second condition (ii) is difficult to verify since *H* and its eigenvalues and eigenvectors generally depend on the current position of $X_{t}$. Instead of evaluating eigenvalues and eigenvectors of *H* and $H^{\prime }$ directly, we use the random matrix property shown in the next theorem.

**Theorem** **5.** *Suppose the upper triangular entries of J follow a probability distribution that is absolutely continuous with respect to the Lebesgue measure. If KerJ={0} is satisfied, then given a point $x\in R^{d}$, λ10(x)≠Reλ1α(x) holds with probability 1.*

The proof is given in Appendix G.1. From this theorem, we simply generate *J* from some probability distribution, such as the Gaussian distribution. Then, we check whether KerJ={0} holds. If KerJ={0} does not hold, we generate a random matrix *J* again.

The above theorem is valid only at a given evaluation point *x*. We can extend the above theorem to all the points over the path of the discretized dynamics (see Appendix G.3). With this procedure, we can theoretically ensure that acceleration occurs with probability one for discretized dynamics.

### 3.4. Qualitative Evaluation of The Acceleration

So far, we have discussed skew acceleration qualitatively but not quantitatively. Although acceleration’s quantitative evaluation is critical for practical purposes, to the best of our knowledge, no existing work has addressed it. In this section, we present a formula that quantitatively assesses skew acceleration by analyzing the eigenvalues of the Hessian matrix.

**Theorem** **6.** *With the identical notation as in Theorem 3, for all x, we have*

(22) Reλ1α(x)=λ10(x)+α2∑k=2dλ10(x)λk0(x)|vk0(x)Jv10(x)|2λk0(x)−λ10(x)+O(α3).

*In particular, at saddle point $x^{*}$, we have*

(23) $$\begin{array}{c} \lambda_{1}^{\alpha }\left(x^{*}\right)=\lambda_{1}^{0}\left(x^{*}\right)+\alpha^{2}\sum_{k=2}^{d}\frac{\lambda_{1}^{0}\left(x^{*}\right)\lambda_{k}^{0}\left(x^{*}\right){\left|v_{k}^{0}\left(x^{*}\right)Jv_{1}^{0}\left(x^{*}\right)\right|}^{2}}{\lambda_{k}^{0}\left(x^{*}\right)-\lambda_{1}^{0}\left(x^{*}\right)}+O\left(\alpha^{3}\right). \end{array}$$

The proofs are shown in Appendix H. When focusing on Equation (22), if U(x) is a strongly convex function, since for all k>1, $\lambda_{k}\left(x\right)>\lambda_{1}\left(x\right)>0$ holds and the second term in Equation (22) is positive. From this, Reλ1α(x)>λ10(x) holds. A similar relation holds for Re(λdα(x)). In Equation (23), $\lambda_{1}^{\alpha }\left(x^{*}\right)<\lambda_{1}^{0}\left(x^{*}\right)<0$ holds. Thus, the changes of the Poincaré constants are proportional to $\alpha^{2}$. With these formulas, we can quantitatively evaluate the acceleration. We present numerical experiments to confirm our theoretical findings in Section 6.1.

## 4. Practical Algorithm for Skew Acceleration

In this section, we discuss skew acceleration in more practical settings compared to Section 3. First, we discuss the memory issue for storing *J* and the discretization of SDE and the stochastic gradient, which are widely used techniques in Bayesian inference. Finally, we present a practical algorithm for skew acceleration.

### 4.1. Memory Issue of Skew Acceleration and Ensemble Sampling

For *d*-dimensional Bayesian models, we need $O(d^{2})$ memory space to store skew matrices *J*s, and this is difficult for high-dimensional models. Instead of storing *J*, we can randomly generate *J*s at each time step following Theorem 5. However, we experimentally confirmed that using different *J*s at each step does not accelerate the convergence (see Section 6). Thus, we need to use a fixed *J* during the iterations.

As discussed below, we found that the previously proposed accelerated parallel sampling [18] can be a practical algorithm to resolve this memory issue. In that method, we simultaneously updated *N* samples of the model’s parameters with correlation. In such a parallel sampling scheme, a correlation exists among multiple Markov chains, it is more efficient than a naive parallel-chain MCMC, where the samples are independent. We express the *n*-th sample at time *t* as $X_{t}^{\left(n\right)}\in R^{d}$ and the joint state of all samples at time *t* as $X_{t}^{⊗N}:={\left(X_{t}^{\left(1\right)},\ldots ,X_{t}^{\left(N\right)}\right)}^{⊤}\in R^{dN}$. We express the joint stationary measure as $\pi^{⊗N}:=\pi ⊗\cdots ⊗\pi \left(x^{⊗N}\right)\propto e^{-\beta \sum_{i=1}^{N}U\left(x^{\left(i\right)}\right)}$. We express the sum of the potential function as $U^{⊗N}:=\sum_{i=1}^{N}U\left(x^{\left(i\right)}\right)$. We then consider the following dynamics:

(24) $$\begin{array}{cc} \; & dX_{t}^{⊗N}\;=\;-\left(I_{dN}+\alpha J\right)\nabla U^{⊗N}\;\left(X_{t}^{⊗N}\;\right)dt\;+\;\sqrt{2\beta^{-1}}dw_{t},\;\;\; \end{array}$$

(25) $$\begin{array}{cc} \; & \nabla U^{⊗N}\left(X_{t}^{⊗N}\right):={\left(\nabla U\left(X_{t}^{\left(1\right)}\right),\ldots ,\nabla U\left(X_{t}^{\left(N\right)}\right)\right)}^{⊤}. \end{array}$$

We call this dynamics skew parallel LD (S-PLD). *N*-independent parallel LD (PLD) is coupled with the skew matrix. Since each chain in PLD is independent of the other, the Poincaré constant of PLD is also $m_{0}$. Ref. [18] argued that the Poincaré constant of S-PLD, m(α,N), satisfies $m\left(\alpha ,N\right)\geq m_{0}$. This means S-PLD shows faster convergence than PLD. As discussed in Section 3.2, these Poincaré constants are characterized by the smallest eigenvalue of the Hessian matrix $\nabla^{2}U^{⊗N}\left(x^{⊗N}\right)$ and $\left(I_{dN}+\alpha J\right)\nabla^{2}U^{⊗N}\left(x^{⊗N}\right)$ where $x^{⊗N}\in R^{dN}$. We denote these smallest eigenvalues as $\lambda_{1}^{0}\left(x^{⊗N}\right)$ and Reλ1α(x⊗N). As discussed in Section 3.2, acceleration occurs if λ10(x⊗N)≠Reλ1α(x⊗N) is satisfied.

In [18], they failed to specify the choice of *J* whose naive construction of *J* requires $O(d^{2}N^{2})$ memory cost. To reduce the memory cost, we propose the following skew matrix:

(26) $$\begin{array}{c} J:=J_{0}⊗I_{d}, \end{array}$$

where $J_{0}$ is a N×N skew matrix and ⊗ is a Kronecker product. We then have the following lemma:

**Lemma** **1.** *If $J_{0}$ is generated based on Theorem 5 and $\mathrm{Ker}J_{0}=\left\{0\right\}$ is satisfied, then given a point $x^{⊗N}$, J does not satisfy the equality condition in Theorems 3,4, which means λ10(x⊗N)≠Reλ1α(x⊗N) with probability 1.*

See Appendix G.2 for the proof. Thus, from this lemma, we only need to prepare and store $J_{0}$, which requires $O(N^{2})$ memory, which does not depend on *d*. In practical settings, this is a significant reduction of the memory size since the number of parallel chains is smaller than the dimension of models. Please note that we can ensure the acceleration with this *J*.

**Lemma** **2.** *Under Assumptions 1–5, assume J satisfies the condition of Lemma 1. Then S-PLD shows*

(27) $$\begin{array}{c} \chi^{2}\left(\mu_{t}^{\alpha ,⊗N}∥\pi^{⊗N}\right)\leq e^{-\frac{2m(\alpha ,N)}{\beta }t}\chi^{2}\left(\mu_{0}^{⊗N}∥\pi^{⊗N}\right), \end{array}$$

*where $\mu_{t}^{\alpha ,⊗N}$ is the measure at time t induced by S-PLD, and $\mu_{0}^{⊗N}$ is the initial measure defined as the product measure of $\mu_{0}$.*

See Appendix I.1 for the proofs. Thus, combined with Lemma 2, S-PLD converges faster than PLD. We also considered the ensemble version of ULD (parallel ULD (PULD)) and its skew accelerated version:

(28) $$\begin{array}{c} \begin{array}{cc} \; & dX_{t}^{⊗N}=\Sigma^{-1}V_{t}^{⊗N}dt+\alpha_{1}J_{1}\nabla U^{⊗N}\left(X_{t}^{⊗N}\right)dt,\\ \; & dV_{t}^{⊗N}=-\nabla U^{⊗N}\left(X_{t}^{⊗N}\right)dt-\gamma \left(\Sigma^{-1}+\alpha_{2}J_{2}\right)V_{t}^{⊗N}dt+\sqrt{2\gamma \beta^{-1}}dw_{t}, \end{array} \end{array}$$

where $J_{1}$ and $J_{2}\in R^{dN\times dN}$ are real-valued skew-symmetric matrices, and $\alpha_{1}$ and $\alpha_{2}\in R_{+}$ are positive constants and $V_{t}^{⊗N}={\left(V_{t}^{\left(1\right)},\ldots ,V_{t}^{\left(N\right)}\right)}^{⊤}\in R^{dN}$. We refer to this dynamics as skew PULD (S-PULD) whose faster convergence can be assured similar to Lemma 2 as shown in Appendix I.2.

### 4.2. Discussion of the Discretization of SDE and Stochastic Gradient and Practical Algorithm

In this section, we further consider practical settings for S-PLD and S-PULD. We discretize these continuous dynamics, e.g., by the Euler-Maruyama method, and approximate the gradient by the stochastic gradient. Although introducing skew matrices accelerates the convergence of continuous dynamics, it simultaneously increases the discretization and stochastic gradient error, resulting in a trade-off. We present a practical algorithm that controls this trade-off.

#### 4.2.1. Trade-off Caused by Discretization and Stochastic Gradient

We consider the following discretization and stochastic gradient for S-PLD and S-PULD:

(29) $$\begin{array}{c} X_{k+1}^{⊗N}=X_{k}^{⊗N}-h\left(I_{dN}+\alpha J\right)\nabla {\hat{U}}^{⊗N}\;\left(X_{k}^{⊗N}\;\right)+\sqrt{2h\beta^{-1}}\epsilon_{k}, \end{array}$$

and

(30) $$\begin{array}{c} \begin{array}{cc} \; & X_{k+1}^{⊗N}=X_{k}^{⊗N}+\Sigma^{-1}V_{k}^{⊗N}h+\alpha J\nabla {\hat{U}}^{⊗N}\left(X_{k}^{⊗N}\right)h\\ \; & V_{k+1}^{⊗N}=V_{k}^{⊗N}\;-\;\nabla {\hat{U}}^{⊗N}\left(X_{k}^{⊗N}\right)h\;-\;\gamma \Sigma^{-1}V_{k}^{⊗N}h\;+\;\sqrt{2\gamma \beta^{-1}h}\epsilon_{k}, \end{array} \end{array}$$

where $\epsilon_{k}\in R^{dN}$ is a standard Gaussian random vector. $\nabla {\hat{U}}^{⊗N}\;\left(X^{⊗N}\;\right)$ is an unbiased estimator of the gradient $\nabla U^{⊗N}\;\left(X^{⊗N}\;\right)$. We refer to Equation (29) as skew-SGLD and Equation (30) as skew-SGHMC. For skew-SGHMC, we dropped $J_{2}$ of S-PULD to decrease the parameters, shown in Appendix B. Please note that skew-SGLD is the identical as the previous dynamics [18]. We introduce an assumption about the stochastic gradient:

**Assumption 7.**  *(Stochastic gradient) There exists a constant δ∈[0,1) such that*

(31) $$\begin{array}{c} E[∥\nabla \hat{U}\left(x\right)-\nabla U\left(x\right){∥}^{2}]\leq 2\delta \left(M^{2}{∥x∥}^{2}+B^{2}\right). \end{array}$$

Given a test function *f* with $L_{f}$ lipschitzness, we approximate ∫fdπ by skew-SGLD or skew-SGHMC, with estimator $\frac{1}{N}\sum_{n=1}^{N}f\left(X_{k}^{\left(n\right)}\right)$. The bias of skew-SGLD is upper-bounded as

**Theorem** **7.** *Under Assumptions 1–7, for any k∈N and any $h\in (0,1\wedge \frac{m}{4M^{2}})$ obeying kh≥1 and βm≥2, we have*

(32) $$\begin{array}{cc} \; & \left|E\frac{1}{N}\sum_{n=1}^{N}f\left(X_{k}^{\left(n\right)}\right)-\int_{R^{d}}fd\pi \right|\leq L_{f}\left(\underset{\left(i\right)}{\underbrace{C_{1}\left(\alpha \right)kh}}+\underset{\left(ii\right)}{\underbrace{C_{2}e^{-\beta^{-1}m\left(\alpha ,N\right)kh}}}\right) \end{array}$$

*and $C_{1}$ and $C_{2}$ depends on the constants of Assumptions 1–7, for the details see Appendix J.*

We present a tighter bias bound in Section 4.3 under a stronger assumption. We can show a similar upper bound for the skew-SGHMC using the same proof strategy. This bound resembles of a previous one [18]; ours shows improved dependency on kh. The previous results of [18] are also limited to LD, not including skew-SGHMC.

Please note that (i) corresponds to the discretization and stochastic gradient error and (ii) corresponds to the convergence behavior of S-PLD, which is continuous dynamics. Since $C_{1}\left(\alpha \right)\geq C_{1}\left(\alpha =0\right)$, skew acceleration increases the discretization and stochastic gradient error. On the other hand, since $m\left(\alpha ,N\right)\geq m_{0}$, the convergence of the continuous dynamics is accelerated. Thus, skew acceleration causes a trade-off. When α is sufficiently small, we derive the explicit dependency of α for this trade-off from an asymptotic expansion. Using the quantitative evaluation of skew acceleration in Theorem 6, we obtain

(33) $$\begin{array}{cc} \; & \left|E\frac{1}{N}\sum_{n=1}^{N}f\left(X_{k}^{\left(n\right)}\right)-\int_{R^{d}}fd\pi \right|\leq \underset{\left(i\right)}{\underbrace{(d_{1}\alpha +d_{2}\alpha^{2})kh}}-\underset{\left(ii\right)}{\underbrace{\alpha^{2}d_{0}e^{-\beta^{-1}m_{0}kh}}}+O\left(\alpha^{3}\right)+\mathrm{const}, \end{array}$$

where $d_{0}$ to $d_{2}$ are positive constants obtained by the asymptotic expansion. See Appendix K for the details. In the above expression, (i) and (ii) correspond to (i) and (ii) of Equation (32). Thus, by choosing appropriate α, we can control the trade-off.

#### 4.2.2. Practical Algorithm Controlling the Trade-off

Since calculating the optimal α that minimizes Equation (33) at each step is computationally demanding, we adaptively tune the value of α by measuring the acceleration with kernelized Stein discrepancy (KSD) [22]. Our idea is to update samples under different α and α+η, and compare KSD between the stationary and empirical distributions of these different interaction strengths. Here, $\eta \in R^{+}$ is a small increment of α. We denote the samples at the (k+1)th step, which is obtained by Equation (29) as $X_{k+1,\alpha }^{⊗N}:=X_{k,\alpha }^{⊗N}-h\left(I_{dN}+\alpha J\right)\nabla {\hat{U}}^{⊗N}\;\left(X_{k,\alpha }^{⊗N}\;\right)+\sqrt{2h\beta^{-1}}\epsilon_{k}$, (or (30) as $X_{k+1,\alpha }^{⊗N}:=X_{k}^{⊗N}+\Sigma^{-1}V_{k}^{⊗N}h+\alpha J\nabla {\hat{U}}^{⊗N}\left(X_{k}^{⊗N}\right)h$). We denote the samples, which are obtained by replacing the above α by α+η, as $X_{k+1,\alpha +\eta }^{⊗N}$. We denote the KSD between the measure of $X_{k+1,\alpha }^{⊗N}$ and stationary measure π as KSD(k+1,α) and estimate the differences of empirical KSD:

(34) $$\begin{array}{cc} \; & \Delta :=\hat{KSD}\left(k+1,\alpha \right)-\hat{KSD}\left(k+1,\alpha +\eta \right), \end{array}$$

where KSD is estimated by

(35) $$\begin{array}{cc} \; & \hat{KSD}\left(k,\alpha \right)=\frac{1}{N(N-1)}\sum_{i=1}^{N}u_{q}\left(X_{k,\alpha }^{\left(i\right)},X_{k,\alpha }^{\left(j\right)}\right), \end{array}$$

(36) $$\begin{array}{cc} \; & u_{q}\left(x,x^{\prime }\right):=\nabla_{x}\log\pi {\left(x\right)}^{⊤}l\left(x,x^{\prime }\right)\nabla_{x}\log\pi \left(x^{\prime }\right)+\nabla_{x}\log\pi {\left(x\right)}^{⊤}\nabla_{x^{\prime }}l\left(x,x^{\prime }\right)\\ \; & \;\;\;\;+\nabla_{x}l{\left(x,x^{\prime }\right)}^{⊤}\nabla_{x}\log\pi +\mathrm{Tr}\nabla_{x,x^{\prime }}l\left(x,x^{\prime }\right), \end{array}$$

where *l* denotes a kernel and we use an RBF kernel. If Δ>0, which indicates that the empirical distribution of $X_{k+1,\alpha +\eta }^{⊗N}$ is closer to the stationary distribution than that of $X_{k+1,\alpha }^{⊗N}$. Thus, we should increase the interaction strength from α to α+η. If Δ<0, we decrease it to α−η. We also update η to cη where c∈(0,1]. The overall process is shown in Algorithm 1. Detailed discussions of the algorithm including how to select $\alpha_{0},\eta_{0}$, and *c* are shown in Appendix L.
**Algorithm 1 **Tuning α**Input: **$X_{k}^{⊗N},\eta_{k},\alpha_{k},c$ **Output: **$\alpha_{k+1},\eta_{k+1}$   1:Calculate $X_{k+1,\alpha_{k}}^{⊗N}$ and $X_{k+1,\alpha_{k}+\eta_{k}}^{⊗N}$.  2:Calculate $\Delta :=\hat{KSD}\left(k+1,\alpha_{k}\right)-\hat{KSD}\left(k+1,\alpha_{k}+\eta_{k}\right)$  3:**if **Δ>0** then**  4: Update $\alpha_{k+1}=\alpha_{k}+\eta_{k}$  5: Update $\eta_{k+1}=\eta_{k}$  6:**else**  7: Update $\alpha_{k+1}=\left|\alpha_{k}-\eta_{k}\right|$  8: Update $\eta_{k+1}=c\eta_{k}$  9:**end if**

Finally, we present Algorithm 2, which describes the whole process. We update the value of α once every $k^{\prime }$ step. Please note that its computational cost is not much larger than that of Equation (30). We only calculate the eigenvalues of *J* once, which requires $O(N^{3})$. The calculation of different KSDs is computationally inexpensive since we can re-use the gradient, which is the most computationally demanding part.
**Algorithm 2 **Proposed algorithm**Input: **$X_{0}^{⊗N},h,\alpha_{0},\eta ,k^{\prime },K,c,\left(V_{0}^{⊗N},\gamma ,\Sigma^{-1}\right)$ **Output: **$X_{K}^{⊗N}$   1:Make a N×N random matrix $J_{0}$ and check $\ker J_{0}=\left\{0\right\}$  2:Set $J=J_{0}⊗I_{d}$  3:**for** k=0 to *K* **do**  4: **if** $\lfloor \frac{k}{k^{\prime }}\rfloor =0$ **then**  5:  Update α by Algorithm 1  6: **end if**  7: Update $X_{k}^{⊗N}$ by Equation (29) (for skew-SGLD)  8: (Update $\left(X_{k}^{⊗N},V_{k}^{⊗N}\right)$ by Equation (30) for skew-SGHMC)  9:**end for**

### 4.3. Refined Analysis for the Bias of Skew-SGLD

When using a constant step size for skew-SGLD, the bound in Theorem 7 is meaningless since the first term of Equation (32) will diverge. Here, following [23], we present a tighter bound for the bias of skew-SGLD under a stronger assumption.

**Theorem** **8.** *Under Assumptions 1–7, for any k∈N and any $h\in (0,1\wedge \frac{\lambda (\alpha ,N)}{4\sqrt{2}M^{2}}\wedge \frac{m}{4M^{2}})$ obeying kh≥1 and βm≥2, we have*

(37) $$\begin{array}{cc} \; & \left|E\frac{1}{N}\sum_{n=1}^{N}f\left(X_{k}^{\left(n\right)}\right)-\int_{R^{d}}fd\pi \right|\leq L_{f}\sqrt{\frac{2}{\lambda (\alpha ,N)}}\sqrt{e^{-\lambda (\alpha ,N)kh}\mathrm{KL}\left(\mu_{0}|\pi \right)+\frac{C_{3}\left(\alpha \right)}{\lambda (\alpha ,N)}}, \end{array}$$

*where*

(38) $$\begin{array}{c} \lambda \left(\alpha ,N\right):={\left(\frac{1}{\left(1+m{\left(\alpha ,N\right)}^{-1}\beta C\left(m_{0}\right)\right)2\pi e^{2}}+\frac{3}{2}m{\left(\alpha ,N\right)}^{-1}\right)}^{-1} \end{array}$$

*and constants $C_{3}\left(\alpha \right)$ and $C(m_{0})$ depend on the constants of Assumptions 1–7. Moreover, λ(α,N) satisfies λ(α,N)≥λ(α=0,N). For the details, see Appendix M.*

Proof is shown in Appendix M. Please note that even if we use a constant step size for skew-SGLD, the bound in Theorem 8 will not diverge. Here we need the stronger assumption about a step size compared to Theorem 7. From Equation (37), the convergence behavior is characterized by λ(α,N) and the bias bound become smaller when λ(α,N) become larger. From the definition of λ(α,N), the larger m(α,N) is, the larger λ(α,N) we obtain. Thus, as we had seen so far, introducing the skew matrices leads to the larger Poincaré constant, and thus, this leads to larger λ(α,N).

Previous work [18] clarified that if α is sufficiently small, introducing skew matrices improves the Poincaré constant by a constant factor, which means that we have $m\left(\alpha ,N\right)-m_{0}\approx O\left(\alpha^{2}\right)$, where $O(\alpha^{2})$ depends on the eigenvector and eigenvalues of the generator L. On the other hand, from Theorem 8, for any ξ>0, to achieve the bias smaller than ξ, it suffice to run skew-SGLD at least for $k\geq \frac{2}{\lambda (\alpha ,N)h}\ln\frac{L_{f}}{\xi }\sqrt{\frac{\mathrm{KL}(\mu_{0}|\pi )}{2\lambda (\alpha ,N)}}$ iterations using the appropriate step size *h* and under the assumption that δ and α are small enough (see Appendix M.2 for details). Combined with these observations, introducing skew matrices into SGLD improves the computational complexity for a constant order. Our numerical experiments show that even constant improvement results in faster convergence in practical Bayesian models.

## 5. Related Work

In this section, we discuss the relationship between our method and other sampling methods.

### 5.1. Relation to Non-Reversible Methods

As we discussed in Section 1, our work extends the existing analysis of non-reversible dynamics [8,18] and presents a practical algorithm. Compared to those previous works, we focus on the practical setting of Bayesian sampling and derive the explicit condition about *J* for acceleration. We also derived a formula to quantitatively evaluate skew acceleration based on the asymptotic expansion of the eigenvalues of the perturbed Hessian matrix. A previous work [24], which derived the optimal skew matrices when the target distribution is Gaussian, requires $O(d^{3})$ computational cost to derive optimal skew matrices, and it is unclear whether it works for non-convex potential functions. On the other hand, our construction method for skew matrices is simple, computationally cheap, and can be applied to general Bayesian models.

Our work analyzes skew acceleration for ULD, which is more effective than LD in practical problems. Another work [8,18] only analyzed skew acceleration for LD. A previous work [17] combined a non-reversible drift term with ULD. Unlike our method, this work’s purpose was to reduce the asymptotic variance of the expectation of a test function and is mainly focusing on sampling from Gaussian distribution.

To the best of our knowledge, our work is the first to focus on the memory issue of skew acceleration and develop a memory-efficient skew matrix for ensemble sampling. Our work also presents an algorithm that controls the trade-off for the first time. Another work [18] identified the trade-off and handled it by cross-validation, which is computationally inefficient, unfortunately.

Finally, we point out an interesting connection between our skew-SGHMC and the magnetic HMC (M-HMC) [25]. M-HMC accelerates HMC’s mixing time by introducing a “magnetic” term into the Hamiltonian. That magnetic term is expressed by special skew matrices. Although a previous work [25] argued that M-HMC is numerically superior to a standard HMC, its theoretical property remains unclear. Thus, our work can analyze the theoretical behavior of magnetic HMC.

### 5.2. Relation to Ensemble Methods

Our proposed algorithm is based on ensemble sampling [26]. Ensemble sampling, in which multiple samples are simultaneously updated with interaction, has been attracting attention numerically and theoretically because of improvements in memory size, computational power, and parallel processing computation schemes [26]. There are successful, widely used ensemble methods, including SVGD [27] and SPOS [28], with which we compare our proposed method numerically in Section 6. Although both show numerically good performance, it is unclear how the interaction term theoretically accelerates the convergence since they are formulated as a McKean–Vlasov process, which is non-linear dynamics, complicating establishing a finite sample convergence rate. Our algorithm is an extension of another work [18], where the interaction was composed of a skew-acceleration term and can be rigorously analyzed. Compared to that previous work [18], we analyzed skew acceleration, focused on the Hessian matrix, and developed practical algorithms, as discussed in Section 4.2, and derived the explicit condition when acceleration occurs, which was unclear [18].

Another difference among SPOS, SVGD, and [18] is that they use first-order methods; our approach uses the second-order method. Little work has been done on ensemble sampling for second-order dynamics. Recently a second-order ensemble method was proposed [29], based on gradient flow analysis. Although its method showed good numerical performance, its theoretical property for finite samples remains unclear since it proposed a scheme as a finite sample approximation of the gradient flow. In contrast, our proposed method is a valid sampling scheme with a non-asymptotic guarantee.

## 6. Numerical Experiments

The purpose of our numerical experiments is to confirm the acceleration of our algorithm proposed in Section 4 in various commonly used Bayesian models including Gaussian distribution (toy data), latent Dirichlet allocation (LDA), and Bayesian neural net regression and classification (BNN). We compared our algorithm’s performance with other ensemble sampling methods: SVGD, SPOS, standard SGLD, and SGHMC. In all the experiments, the values and the error bars are the mean and the standard deviation of repeated trials. For all the experiments we set γ=1 and $\Sigma^{-1}=300$ for SGHMC and Skew-SGHMC. As for the hyperparameters of our proposed algorithm, the selection criterion is discussed in Appendix L.

### 6.1. Toy Data Experiment

The target distribution is the multivariate Gaussian distribution, π=N(μ,Ω) where we generated $\Omega^{-1}=A^{⊤}A$ and each element of $A\in R^{2d\times d}$ is drawn from the standard Gaussian distribution. The dimension of the target distribution is d=50, we approximate by 20 samples using the proposed ensemble methods. We tested these toy data because the LD for this target distribution is known as the Ornstein–Uhlenbeck process, and its theoretical properties have been studied extensively e.g., [30]. Thus, by studying the convergence behavior of these toy data, we can understand our proposed method more clearly.

First, we confirmed how the skew-symmetric matrix affects the eigenvalues of the Hessian matrix, as discussed in Section 3, where we only showed the asymptotic expansion for the smallest real part of the eigenvalues and saddle point. Here we can show a similar expansion for the largest real part:

(39) ReλdNα=λdN0+α2∑k=1dN−1λdN0λk0|vk0JvdN0|2λk0−λdN0+O(α3).

ReλdNα≤λdNα holds.

**Figure 2:**
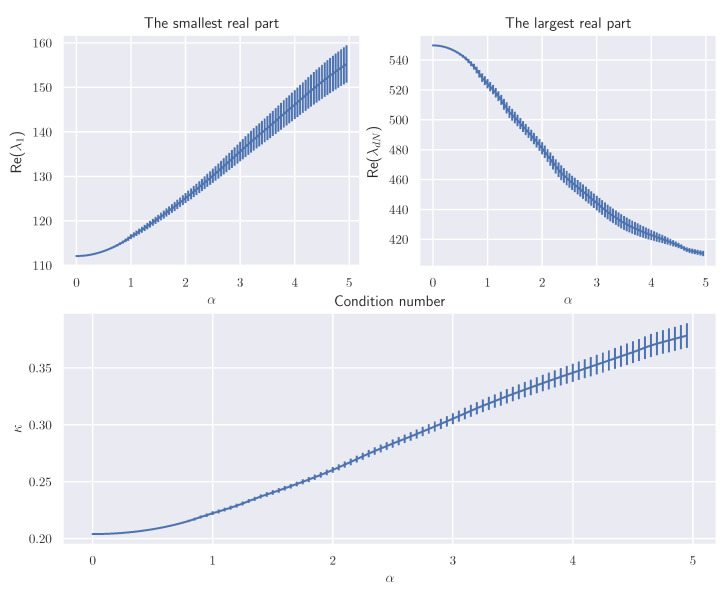
Eigenvalue changes (averaged over ten trials).

Then we observed how the largest and smallest real parts of the eigenvalues of $\left(I+\alpha J\right)\Omega^{-1}$ depend on α. The results are shown in Figure 2, where we averaged 10 trials over a randomly made *J* with fixed *A*. The upper-left, upper-right, and lower figures show Re(λ1(α)), Re(λdN(α)), and Re(λ1(α))/Re(λdN(α)). These behaviors are consistent with Theorem 3. When α is small, its behavior is close to the quadratic function proved in Theorem 3.

**Figure 3:**
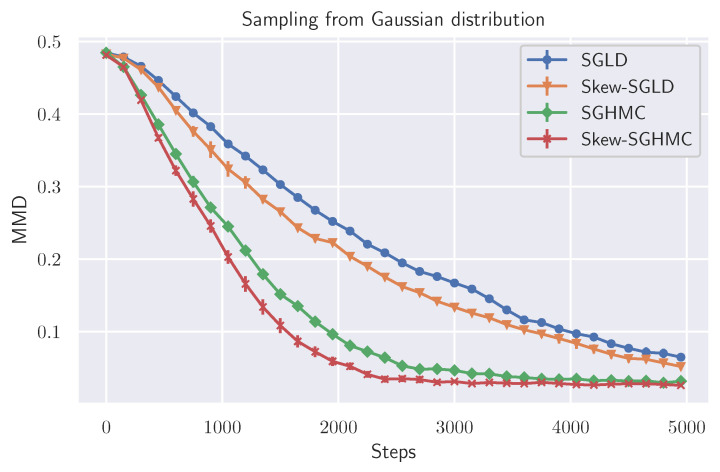
Convergence behavior of toy data in MMD (averaged over ten trials).

Next, we observed the convergence behavior of skew-SGLD and skew-SGHMC. We measured the convergence by maximum mean discrepancy (MMD) [31] between the empirical and stationary distributions. For MMD, we used 2000 samples for the target distribution, and we used the Gaussian kernel whose bandwidth is set to the median distance of these 2000 samples. We used gradient descent (GD), with step size $h=1\times 10^{-4}$. The results are shown in Figure 3. The proposed method shows faster convergence than naive parallel sampling, which is consistent with Table 2.

### 6.2. LDA Experiment

**Figure 4:**
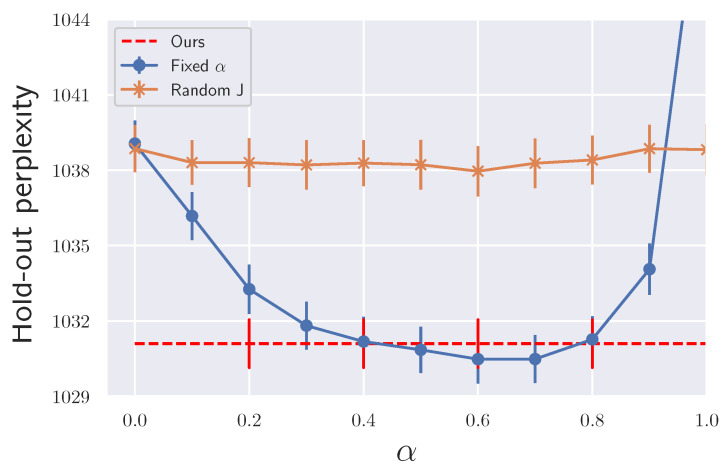
Final performances of LDA under different values of α (averaged over ten trials).

**Figure 5:**
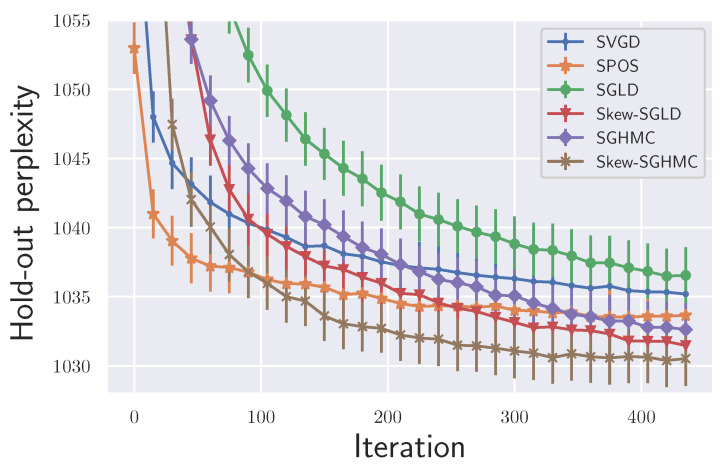
LDA experiments (Averaged over 10 trials).

We tested with an LDA model using the ICML dataset [32] following the same setting as [33]. We used 20 samples for all the methods. Minibatch size is 100. We used step size $h=5\times 10^{-4}$. First, we confirmed the effectiveness of our proposed Algorithm 1, which adaptively tunes α values. For that purpose, we compared the final performance obtained by our methods with a previous method [18], in which α is selected by cross-validation (CV). Here instead of CV, we just fixed α during the sampling and refer to it as fixed α. We also tested the case when *J* is generated randomly at each step with fixed α, as discussed in Section 4.1. We refer to it as random J. The results are shown in Figure 4 where skew-SGLD was used. We found that our method showed competitive performance with the best performance of fixed α. For the computational cost, we used $k^{\prime }=2$ in Algorithm 2, and our method needed twice the wall clock time than each fixed α. This means that our algorithm greatly reduces the total computational time since we tried more than two αs in the fixed α for CV. We also found that since using different *J*s at each step did not accelerate the performance, we need to store and fix *J* during the sampling for acceleration. Next, we compared our method with other ensemble sampling schemes and observed the convergence speed. The result is shown in Figure 5. Skew-SGLD and skew-SGHMC outperformed SGLD and SGHMC, which is consistent with our theory.

### 6.3. BNN Regression and Classification

**Figure 6:**
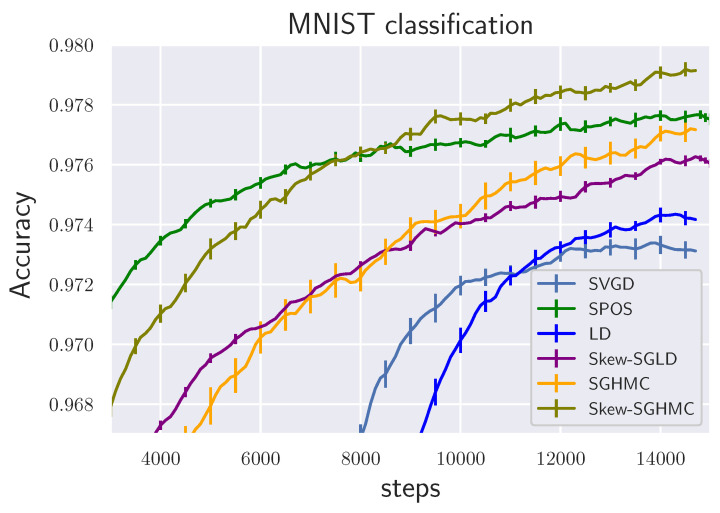
MNIST classification (Averaged over ten trials).

We tested with the BNN regression task using the UCI dataset [34], following a previous setting Liu and Wang [27]. We used one hidden layer neural network model with ReLU activation and 100 hidden units. We used 10 samples for all the methods. We used the minibatch size 100. We used step size $h=5\times 10^{-5}$. The results are shown in Table 1 and Table 2. We also tested on BNN classification task using the MNIST dataset. The result is shown in Figure 6. We used one hidden layer neural network model with ReLU activation and 100 hidden units. Batchsize is 500 and we set step size $h=5\times 10^{-5}$. Our proposed methods outperformed other ensemble methods. Please note that skew-SGHMC and skew-SGLD consistently outperformed SGHMC and SGLD.

## 7. Conclusions

We studied skew acceleration for LD and ULD from practical viewpoints and concluded that the improved eigenvalues of the perturbed Hessian matrix caused acceleration and derived the explicit condition for acceleration. We described a novel ensemble sampling method, which couples multiple SGLD or SGHMC with memory-efficient skew matrices. We also proposed a practical algorithm that controls the trade-off of faster convergence and larger discretization and stochastic gradient error and numerically confirmed the effectiveness of our proposed algorithm.

## Figures and Tables

**Table 1:** Benchmark results on test RMSE for regression task.

Dataset	Avg. Test RMSE					
	SVGD	SPOS	SGLD	Skew-SGLD	SGHMC	Skew-SGHMC
Concrete	5.709 ± 0.040	5.239 ± 0.199	5.009 ± 0.091	4.973 ± 0.057	4.949 ± 0.144	4.790 ± 0.081
Kin8nm	0.0731 ± 0.0006	0.0688 ± 0.0003	0.0693 ± 0.0006	0.0689 ± 0.0005	0.0687 ± 0.0001	0.0683 ± 0.0003
Energy	0.520 ± 0.060	0.456 ± 0.030	0.428 ± 0.045	0.412 ± 0.045	0.406 ± 0.019	0.403 ± 0.008
Bostonhousing	3.306 ± 0.005	3.107 ± 0.173	2.948 ± 0.084	2.930 ± 0.095	3.053 ± 0.093	2.986 ± 0.143
Winequality	0.619 ± 0.001	0.618 ± 0.007	0.641 ± 0.003	0.634 ± 0.004	0.614 ± 0.004	0.613 ± 0.004
PowerPlant	4.219 ± 0.012	4.160 ± 0.009	4.129 ± 0.002	4.118 ± 0.006	4.112 ± 0.009	4.105 ± 0.008
Yacht	0.475 ± 0.049	0.467 ± 0.110	0.464 ± 0.058	0.442 ± 0.046	0.464 ± 0.078	0.432 ± 0.051

**Table 2:** Benchmark results on test negative log likelihood for regression task.

Dataset	Avg. Test Negative Log Likelihood					
	SVGD	SPOS	SGLD	Skew-SGLD	SGHMC	Skew-SGHMC
Concrete	−3.157 ± 0.008	−3.124 ± 0.025	−3.052 ± 0.009	−3.049 ± 0.012	−3.046 ± 0.025	−3.033 ± 0.021
Kin8nm	1.153 ± 0.0084	1.212 ± 0.008	1.223 ± 0.002	1.223 ± 0.005	1.230 ± 0.0015	1.235 ± 0.0025
Energy	−0.816 ± 0.102	−0.976 ± 0.079	−0.867 ± 0.056	−0.845 ± 0.021	−0.843 ± 0.045	−0.844 ± 0.041
Bostonhousing	−2.98 ± 0.000	−2.644 ± 0.027	−2.548 ± 0.016	−2.539 ± 0.002	−2.574 ± 0.019	−2.561 ± 0.017
Winequality	−1.012 ± 0.000	−0.959 ± 0.007	−0.976 ± 0.006	−0.968 ± 0.005	−0.941 ± 0.007	−0.938 ± 0.005
PowerPlant	−2.871 ± 0.004	−2.850 ± 0.004	−2.844 ± 0.002	−2.842 ± 0.001	−2.838 ± 0.004	−2.835 ± 0.003
Yacht	−1.184 ± 0.06	−1.372 ± 0.07	−1.077 ± 0.066	−1.078 ± 0.030	−1.083 ± 0.030	−1.079 ± 0.051

**Figure A1:**
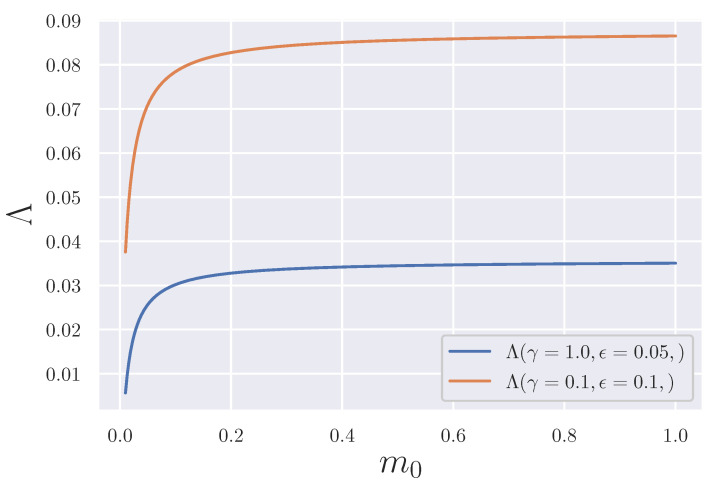
The convergence rate of ULD under the different Poincaré constants.

## Data Availability

Publicly available datasets were analyzed in this study. This data can be found here: http://archive.ics.uci.edu/ml (accessed on 21 June 2021).

## Abbreviations

The following abbreviations are used in this manuscript:

LD	Langevin Dynamics
MCMC	Markov Chain Monte Carlo
ULD	Underdamped Langevin Dynamics
SGLD	Stochastic Gradient Langevin Dynamics
SGHMC	Stochastic Gradient Hamilton Monte Carlo
PLD	Parallel Langevin Dynamics
PULD	Parallel Underdamped Langevin Dynamics
SLD	Skew Langevin Dynamics
S-ULD	Skew Underdamped Langevin Dynamics
S-PLD	Skew Parallel Langevin Dynamics
S-PULD	Skew Parallel Underdamped Langevin Dynamics
KSD	Kernelized Stein Discrepancy

## Appendix A. Additional Backgrounds

We introduce additional backgrounds which are used in our Proof.

### Appendix A.1. Wasserstein Distance and Kullback–Leibler Divergence

In this paper, we use the Wasserstein distance. Let us define the Wasserstein distance. Let (E,d) be a metric space (appropriate space such as Polish space) with σ field A, where d(·,·) is A×A-measurable. Let μ, ν are probability measures on *E*, and p≥1. The Wasserstein distance of order *p* with cost function *d* between μ and ν is defined as

(A1) $$\begin{array}{c} W_{p}^{d}\left(\mu ,\nu \right)=\underset{\pi \in \Pi (\mu ,\nu )}{\inf}{\left(\int \int d{\left(x,y\right)}^{p}d\pi \left(x,y\right)\right)}^{1/p}, \end{array}$$

where Π(μ,ν) is the set of all joint probability measures on E×E with marginals μ and ν. In this paper, we work on the space $R^{d}$. As for the distance, we use the Euclidean distance, ∥·∥. For simplicity, we express the p-Wasserstein distance with the Euclidean distance as $W_{p}$. The various properties of Wasserstein distance are summarized in [35]. We define the Kullback–Leibler (KL) divergence as

(A2) $$\begin{array}{c} \mathrm{KL}\left(\nu ∥\mu \right)=\left\{\begin{array}{c} \int \log\frac{d\nu }{d\mu }d\nu ,\;\;\;\;\nu \ll \mu ,\\ +\infty ,\;\;\;\;\;\;\;\;\;\;\;\mathrm{otherwise}. \end{array}\right. \end{array}$$

### Appendix A.2. Markov Diffusion and Generator

Here we introduce the additional explanation about the generator of the Markov diffusion process. Given an SDE,

(A3) $$\begin{array}{c} dX_{t}=-\nabla U\left(X_{t}\right)dt+\sqrt{2\beta^{-1}}dw\left(t\right), \end{array}$$

and we denote the corresponding Markov semigroup as $P={\left\{P_{t}\right\}}_{t>0}$ and define the Kolmogorov operator as $P_{s}$ which is defined as $P_{s}f\left(X_{t}\right)=E\left[f\left(X_{t+s}\right)|X\left(t\right)\right]$, where $f:R^{d}\to R$ is some bounded test function in $L^{2}\left(\mu \right)$. A property $P_{s+t}=P_{s}\circ P_{t}$ is called Markov property. A probability measure π is the stationary distribution when it satisfies for all measurable bounded function *f* and *t*, $\int_{R^{d}}P_{t}fd\pi =\int_{R^{d}}fd\pi$.

We denote the infinitesimal generator of the associated Markov group as L and we call it a generator for simplicity. The linearity of the operators of $P_{t}$ with the semigroup property indicates that L is the derivative of $P_{t}$ as

(A4) $$\begin{array}{c} \frac{1}{h}\left(P_{t+h}-P_{t}\right)=P_{t}\frac{1}{h}\left(P_{h}-Id\right)=\frac{1}{h}\left(P_{h}-Id\right)P_{t}, \end{array}$$

where Id is the identity map. In addition, taking h→0, we have $\partial P_{t}=LP_{t}=P_{t}L$. From the Hille–Yoshida theory [19], there exists a dense linear subspace of $L^{2}\left(\pi \right)$ on which L exists. We refer it as D(L). If the Markov semigroup is associated with the SDE of Equation (A3), the generator can be written as

(A5) $$\begin{array}{cc} Lf(X_{t}): & =\lim_{h\to 0^{+}}\frac{E\left(f\left(X_{t+h}\right)|X_{t}\right)-f\left(X_{t}\right)}{h}=\left(-\nabla U\left(X_{t}\right)\cdot \nabla +\beta^{-1}\Delta \right)f\left(X_{t}\right), \end{array}$$

where Δ is the Laplacian in the standard Euclidean space. The generator satisfies $L1=0,\;\int_{R^{d}}Lfd\pi =0$.

### Appendix A.3. Poincaré Inequality

We use the Poincaré inequality to measure the speed of convergence to the stationary distribution. In this section, we summarize definitions and useful properties of them and see [19] for more details. We define the Dirichlet form E(f) for all bounded functions f∈D(L) where D(L) denotes the domain of L as

(A6) $$\begin{array}{c} E\left(f\right):=-\int_{R^{d}}fLfd\pi . \end{array}$$

E(f)>0 is satisfied. By the partial integration, we have $E\left(f\right)=-\int_{R^{d}}fLfd\pi =\frac{1}{\beta }\int_{R^{d}}{∥\nabla f∥}^{2}d\pi$. We define a Dirichlet domain, D(E), which is the set of functions $f\in L^{2}\left(\pi \right)$ and satisfies E(f)<∞.

We say that π with L satisfies *a Poincaré inequality* with a positive constant *c* if for any f∈D(E), π with L satisfies,

(A7) $$\begin{array}{c} \int f^{2}d\pi -{\left(\int fd\pi \right)}^{2}\leq cE\left(f\right). \end{array}$$

This constant *c* is closely related to a spectral gap. If the smallest eigenvalue of L, λ, is greater than 0, then it is called the spectral gap. If the spectral gap λ>0 exists, then it is written as

(A8) $$\begin{array}{c} \lambda :=\inf_{f\in D(E)}\left\{\frac{E(f)}{\int f^{2}d\pi }:f\neq 0,\int fd\pi =0\right\}. \end{array}$$

From this, a constant *c* which satisfies c≥1/λ, can also satisfy the Poincaré inequality. To check the existence of the spectral gap, one approach is to use the Lyapunov function, which is developed by Bakry et al. [36].

We can also express the Poincaré inequality via chi divergence. Let us define the $\chi^{2}$ divergence for μ≪π as

(A9) $$\begin{array}{c} \chi^{2}\left(\mu ∥\pi \right):={∥\frac{d\mu }{d\pi }-1∥}_{L_{\pi }^{2}}^{2}=\int_{R^{d}}{\left|\frac{d\mu }{d\pi }-1\right|}^{2}d\pi . \end{array}$$

Then, we express the Poincaré inequality with a constant *c* for all μ≪π as

(A10) $$\begin{array}{c} \chi^{2}\left(\mu ∥\pi \right)\leq c\;E\left(\sqrt{\frac{d\mu }{d\pi }}\right). \end{array}$$

We obtain the following exponential convergence results from the above functional inequalities for measures.

**Theorem A1.** 
*(Exponential convergence in the variance, Theorem 4.2.5 in [19]) When π satisfies the Poincaré inequality with a constant c, it implies the exponential convergence in the variance with a rate 2/c, i.e., for every bounded function $f:R^{d}\to R$,*

(A11) $$\begin{array}{c} {\mathrm{Var}}_{\pi }\left(P_{t}f\right)\leq e^{-2t/c}{\mathrm{Var}}_{\pi }\left(f\right), \end{array}$$

*where ${\mathrm{Var}}_{\pi }\left(f\right):=\int_{R^{d}}f^{2}d\pi -{\left(\int_{R^{d}}fd\pi \right)}^{2}$.*

We also introduce the important property of Poincaré inequality as for the product measures. These relations play important roles in our analysis.

**Theorem A2.** 
*(Stability under the product, Proposition 4.3.1 in [19]) If $\mu_{1}$ and $\mu_{2}$ on $R^{d}$ satisfy the Poincaré inequalities with a constant $c_{1}$ and $c_{2}$, then the product $\mu_{1}⊗\mu_{2}$ on $R^{d}⊗R^{d}$ satisfies the Poincaré inequality with the constant $\max(c_{1},c_{2})$.*

## Appendix B. Generator of the Underdamped Langevin Dynamics (ULD)

Following [10], we define the infinitesimal generator of the ULD as

(A12) $$\begin{array}{cc} \; & Lf\left(x,v\right):=-\left(\gamma v+\nabla U\left(x\right)\right)\nabla_{v}f\left(x,v\right)+\gamma \beta^{-1}\Delta f\left(x,v\right)+v\nabla_{x}f\left(x,v\right). \end{array}$$

Then, we define the generator of S-ULD as

(A13) $$\begin{array}{cc} Lf(x,v):=- & \left(\gamma v+\nabla U(x\right))\nabla_{v}f\left(x,v\right)+\gamma \beta^{-1}\Delta f\left(x,v\right)\\ \; & +v\nabla_{x}f\left(x,v\right)+\alpha_{1}J_{1}\nabla U\left(x\right)\nabla_{x}f\left(x,v\right)+\alpha_{1}J_{2}\Sigma^{-1}v\nabla_{v}f\left(x,v\right), \end{array}$$

where the second line corresponds to the interaction terms. Then it is easily to confirm $\int_{R^{2d}}Lf\left(x,v\right)d\tilde{\pi }=0$, where $\tilde{\pi }:=\pi ⊗N\left(0,\Sigma \right)\propto e^{-\beta U\left(x\right)-\frac{1}{2}\Sigma^{-1}{∥v∥}^{2}}$. Thus, the stationary distribution of S-ULD is $\tilde{\pi }$. We can prove this by simply using the partial integral and using the property of the skew-symmetric matrix. Thus, the stationary distribution of S-ULD is $\tilde{\pi }$.

We consider other combinations the skew matrices with ULD. For example, we can consider the following more general combination;

(A14) $$\begin{array}{c} \begin{array}{cc} \; & dX_{t}=\Sigma^{-1}V_{t}dt+\alpha_{1}J_{1}\nabla U\left(X_{t}\right)dt+\alpha_{2}\Sigma^{-1}J_{2}V_{t}dt\\ \; & dV_{t}=-\nabla U\left(X_{t}\right)dt-\gamma \Sigma^{-1}V_{t}dt+\alpha_{3}J_{3}V_{t}dt+\alpha_{4}J_{4}\nabla U\left(X_{t}\right)dt+\sqrt{2\gamma \beta^{-1}}dw_{t}, \end{array} \end{array}$$

compared to S-ULD, there are new two terms are included. We can also derive the infinitesimal generator of this Markov process. We express it as $\tilde{L}$. Then we calculate the infinitesimal change of the expectation of *f*

(A15) $$\begin{array}{c} \int_{R^{2d}}\tilde{L}f\left(x,v\right)d\tilde{\pi }\neq 0, \end{array}$$

which suggests that the stationary distribution of Equation (A14) is different form $\tilde{\pi }$.

It is widely known that underdamped Langevin dynamics converges to (overdamped) Langevin dynamics. Here we observe that S-ULD converges to Skew-LD in [18]. The limiting procedure is widely known, for example, see [17,37,38]. We cite Proposition 1 in [17]; given a stochastic process

(A16) $$\begin{array}{c} \begin{array}{cc} \; & dX_{t}=\Sigma^{-1}V_{t}dt+\alpha_{1}J_{1}\nabla U\left(X_{t}\right)dt,\\ \; & dV_{t}=-\nabla U\left(X_{t}\right)dt-\gamma \Sigma^{-1}V_{t}dt-\alpha_{2}\Sigma^{-1}J_{2}V_{t}dt+\sqrt{2\gamma }dw_{t}, \end{array} \end{array}$$

and we rescale it by introducing ϵ which expresses the small mass limit as

(A17) $$\begin{array}{c} \begin{array}{cc} \; & dX_{t}=\frac{1}{\epsilon }\Sigma^{-1}V_{t}dt+\alpha_{1}J_{1}\nabla U\left(X_{t}\right)dt,\\ \; & dV_{t}=-\frac{1}{\epsilon }\nabla U\left(X_{t}\right)dt-\frac{1}{\epsilon^{2}}\gamma \Sigma^{-1}V_{t}dt-{\frac{1}{\epsilon }}^{2}\alpha_{2}\Sigma^{-1}J_{2}V_{t}dt+\frac{1}{\epsilon }\sqrt{2\gamma }dw_{t}, \end{array} \end{array}$$

and by taking the limit ϵ→0, the dynamics converges to

(A18) $$\begin{array}{c} dX_{t}=-{\left(\alpha_{2}J_{2}+\gamma \right)}^{-1}\nabla U\left(X_{t}\right)dt-\alpha_{1}J_{1}\nabla U\left(X_{t}\right)+{\left(\alpha_{2}J_{2}+\gamma \right)}^{-1}\sqrt{2\gamma }dw_{t}. \end{array}$$

See Proposition 1 in [17], for the precise statements. Please note that the term related $J_{2}$ works as preconditioning. Thus, if we set $\alpha_{2}J_{2}=0$, the obtained dynamics are equivalent to the continuous dynamics of skew-SGLD. Thus, our skew-SGHMC is the natural extension of skew-SGLD.

## Appendix C. Proof of Theorem 1

### Appendix C.1. Proof for S-LD

First, under Asuumptions 1–5, LD has a spectral gap, and its Poincaré constant is upper bounded as

(A19) $$\begin{array}{c} \frac{1}{m_{0}}\leq \frac{2C(d+b\beta )}{m\beta }\exp\left(\frac{2}{m}\left(M+B\right)\left(b\beta +d\right)+\beta \left(A+B\right)\right)+\frac{1}{m\beta (d+b\beta )}. \end{array}$$

and this is derived in [2].

Next, we introduce the generator of S-LD

$$\begin{array}{c} L_{\alpha }f\left(x\right)=\left(-\nabla U_{\alpha }\left(x\right)\cdot \nabla +\beta^{-1}\Delta \right)f\left(x\right), \end{array}$$

where $\nabla U_{\alpha }\left(x\right):=\nabla U\left(x\right)+\alpha J\nabla U\left(x\right)$.

The proof is almost similar to [18] of Theorem 12.

**Proof of Theorem 1.** Since the generator $L_{\alpha =0}$ is self-adjoint, and the suitable growth condition, the spectral of $L_{\alpha =0}$ is discrete [19]. We denote the spectrum of $L_{\alpha =0}$ as ${\left\{\lambda_{k}\right\}}_{k=0}^{\infty }\in R$ and corresponding normalized eigenvectors as ${\left\{e_{k}\right\}}_{k=0}^{\infty }$, which are the real functions. We order the spectrum as $0>\lambda_{0}>\lambda_{1}>\ldots$. Thus, $m_{0}=-\lambda_{0}$. As for $L_{\alpha }$, although it is not a self-adjoint operator, from Proposition 1 in Franke et al. [39], it has discrete complex spectrums. We denote the spectrum of $L_{\alpha }$ as λ+iμ∈C where λ,μ∈R and corresponding normalized eigenvector as u+iv where u,v are the real functions and then we have

(A20) $$\begin{array}{c} L_{\alpha }\left(u+iv\right)=\left(\lambda +i\mu \right)\left(u+iv\right). \end{array}$$

From this definition, by checking the real parts and complex parts, following relations are derived

(A21) $$\begin{array}{c} L_{\alpha }u=\lambda u-\mu v, \end{array}$$

(A22) $$\begin{array}{c} L_{\alpha }v=\lambda v+\mu u. \end{array}$$

Due to the divergence-free drift property, for any bounded real value test function g(x),

(A23) $$\begin{array}{cc} \int g(L_{\alpha =0}-L_{\alpha })gd\pi & =\int \alpha g\gamma \cdot \nabla gd\pi =-\int \alpha g\gamma \cdot \nabla gd\pi , \end{array}$$

where we used the partial integral. This means that for any bounded real function g(x),

(A24) $$\begin{array}{c} \int gL_{\alpha =0}gd\pi =\int gL_{\alpha }gd\pi . \end{array}$$

(This only holds for real functions.) Then, we can evaluate the real part of the eigenvalue λ as follows,

(A25) $$\begin{array}{cc} \int uL_{\alpha =0}ud\pi +\int vL_{\alpha =0}vd\pi & =\int uL_{\alpha }ud\pi +\int vL_{\alpha }vd\pi =\lambda \left(\int u^{2}d\pi +\int v^{2}d\pi \right)=\lambda . \end{array}$$

Then, by expanding the eigenfunction u,v by the eigenfunction $\left\{e_{k}\right\}$,

(A26) $$\begin{array}{cc} \lambda =\int uL_{\alpha =0}ud\pi +\int vL_{\alpha =0}vd\pi & =\sum_{k}\lambda_{k}\left({\left(\int ue_{k}d\pi \right)}^{2}+{\left(\int ve_{k}d\pi \right)}^{2}\right)\\ \; & \leq \lambda_{0}\sum_{k}\left({\left(\int ue_{k}d\pi \right)}^{2}+{\left(\int ve_{k}d\pi \right)}^{2}\right)\leq \lambda_{0}. \end{array}$$

Thus, the real part of the eigenvalue of $L_{\alpha }$ is smaller than the smallest eigenvalue of $L_{\alpha }$. This means that the spectral gap of $L_{\alpha }$ is larger than that of $L_{\alpha =0}$, i.e., $m\left(\alpha \right)\geq m_{0}$ holds. □

### Appendix C.2. Proof of Theorem 2 (S-ULD)

**Proof of Theorem 2.** To prove the S-ULD, we use the result of [20], which characterize the convergence of ULD via the Poincaré constant. Let us denote ${\tilde{\mu }}_{t}$ as the measure induced by ULD. Then from Theorem 1 of [20], if π with L has the Poincaré constant $m_{0}$, we have

(A27) $$\begin{array}{c} \chi^{2}\left({\tilde{\mu }}_{t}∥\tilde{\pi }\right)\leq \frac{1+\bar{\epsilon }}{1-\bar{\epsilon }}e^{-\lambda_{\gamma }t}\chi^{2}\left({\tilde{\mu }}_{t}∥\tilde{\pi }\right). \end{array}$$

where $\bar{\epsilon }$ and $\lambda_{\gamma }$ is given as follows.

(A28) $$\begin{array}{c} \lambda_{\gamma }=\frac{\Lambda (\gamma ,\bar{\epsilon }\min\left(\gamma ,\gamma^{-1}\right))}{1+\bar{\epsilon }\min\left(\gamma ,\gamma^{-1}\right)}, \end{array}$$

where

(A29) $$\begin{array}{cc} \; & \Lambda \left(\gamma ,\epsilon \right)=\frac{\gamma \Sigma^{-1}-\frac{1}{1+\frac{m_{0}\Sigma^{-1}}{\beta }}}{2}-\frac{1}{2}\sqrt{{\left(S_{--}-S_{++}\right)}^{2}+{\left(S_{-+}\right)}^{2}}, \end{array}$$

(A30) $$\begin{array}{cc} \; & S_{--}=\epsilon \lambda_{ham}, \end{array}$$

(A31) $$\begin{array}{cc} \; & S_{-+}=-\epsilon \left(R_{ham}+\gamma \Sigma^{-1}/2\right), \end{array}$$

(A32) $$\begin{array}{cc} \; & S_{++}=\gamma \Sigma^{-1}-\epsilon , \end{array}$$

(A33) $$\begin{array}{cc} \; & \lambda_{ham}=1-{\left(1+\frac{m_{0}\Sigma^{-1}}{\beta }\right)}^{-1}, \end{array}$$

(A34) $$\begin{array}{cc} \; & \epsilon =\bar{\epsilon }\min\left(\gamma ,\gamma^{-1}\right), \end{array}$$

where $\bar{\epsilon }$ is arbitrary sufficiently small positive value such that $\Lambda (\gamma ,\bar{\epsilon }\min\left(\gamma ,\gamma^{-1}\right))>0$ is satisfies. As for $R_{ham}$, if there exists a positive constant *K*, such that $\nabla^{2}U\geq -KI$, then $R_{ham}\leq \sqrt{\max\{K,2\}}$. In our assumption, this corresponds to βM, thus $R_{ham}\leq \sqrt{\max\{\beta M,2\}}$. From the above definitions, we can see that the larger $m_{0}$ is, i.e., the larger the Poincaré constant is the faster convergence ULD shows. This can also be confirmed numerically, see Figure A1, which shows how the Λ changes under different $m_{0}$. We set $\Sigma^{-1}=100$. From the figure, the larger the Poincaré constant is, the larger Λ becomes.

So far, we confirmed that the convergence speed of S-ULD is characterized by the Poincaré constant of L. When we consider S-ULD, we simply add the skew matrices term to the generator of the ULD in the proof of Proposition 1 in [20]. This means that we simply replace the Poincaré constant from $m_{0}$ to m(α) in the proof of Proposition 1 in [20]. Then, $m_{0}$ will be replaced with m(α) that indicates the faster convergence. □

## Appendix D. Eigenvalue and Poincaré Constant

In this section, we discuss the relation between eigenvalues of the Hessian matrix and Poincaré constant.

### Appendix D.1. Strongly Convex Potential Function

When we consider LD with *m*-strongly convex potential function, then the Poincaré constant is *m*, this means exponential convergence with rate *m* (See [19] for the detail).

We then consider the S-LD with *m*-strongly convex function. In this setting, by considering the synchronous coupling technique [11], we can show that the variance decays exponentially with the rate of the smallest real part of the eigenvalue. This is because that by preparing two S-LD $\left(X_{t},Y_{t}\right)$ given as

(A35) $$\begin{array}{c} dX_{t}=-\left(I+\alpha J\right)\nabla U\left(X_{t}\right)dt+\sqrt{2\beta^{-1}}dw_{t},\;\;dY_{t}=-\left(I+\alpha J\right)\nabla U\left(Y_{t}\right)dt+\sqrt{2\beta^{-1}}dw_{t}^{\prime }. \end{array}$$

Then we evaluate the behavior of $∥X_{t}-Y_{t}{∥}^{2}$. From Ito lemma and considering the synchronous coupling, we obtain

(A36) $$\begin{array}{c} \frac{d}{dt}∥X_{t}-Y_{t}{∥}^{2}=-\left(X_{t}-Y_{t}\right)\cdot \frac{\left(I+\alpha J\right)}{\beta }\left(\nabla U\left(X_{t}\right)-\nabla U\left(Y_{t}\right)\right)\leq -\frac{2m(\alpha )}{\beta }{∥X_{t}-Y_{t}∥}^{2}, \end{array}$$

where m(α) is the constant that satisfies m(α)≤Reλ1α(x) for all *x*, see Appendix E for details. This means that variance decays exponentially with the rate $\frac{2m(\alpha )}{\beta }$. From the fundamental property of the Poincaré constant (Theorem 4.2.5 in [19]), m(α) is the Poincaré constant. Thus the imaginary part has no effect on the continuous dynamics. Thus, the Poincaré inequality is the smallest real part of the perturbed Hessian matrix.

### Appendix D.2. Non-Convex Potential Function

As we discussed in Section 3.1, [21] derived the sharper estimation for the Poincaré constant for the non-convex potential function. It is easy to verify that their assumptions are satisfied under our assumption 1–5. Following the main paper, we denote $x_{1}$ global minima, and $x_{2}$ is the local minima which have the second smallest value in U(x). We express the saddle point between $x_{1}$ and $x_{2}$ as $x^{*}$. To be more precise, the saddle point that characterizes the Poincaré constant is known as the critical point with index one defined as

(A37) $$\begin{array}{c} U\left(x^{*}\right)=\inf\left\{\max_{s\in [0,1]}U\left(\gamma \left(s\right)\right):\gamma \in C\left(\left[0,1\right],R^{d}\right),\gamma \left(0\right)=x_{1},\gamma \left(1\right)=x_{2}\right\}, \end{array}$$

and the eigenvalue of $\nabla^{2}U\left(x^{*}\right)$ has one negative eigenvalue and d−1 positive eigenvalues. We express them as $\lambda_{1}\left(x^{*}\right)<0<\lambda_{2}\left(x^{*}\right)<\ldots ,\lambda_{d}\left(x^{*}\right)$.

Ref. [21] studied the Poincaré constant by decomposing the non-convex potential focusing on attractors. By focusing on attractors, they showed that the non-convex potential can be decomposed into the sum of *approximately* Gaussian distributions. They proved that the Poincaré constant is characterized by the local Poincaré constants, these are derived by the approximate Gaussian distribution on the attractors and their surrounding regions. In addition, they proved that the dominant term of the Poincaré constant is specified by the saddle points between the global minima and the point which takes the second smallest value for U(x). From Theorem 2.12 and Corollary 2.15 in [21], the Poincaré constant is characterized by

(A38) $$\begin{array}{c} m_{0}^{-1}\approx \frac{\sqrt{\det H(x^{*})}}{\sqrt{Z|\lambda_{1}\left(x^{*}\right)|\det H\left(x_{1}\right)}\sqrt{\det H(x_{2})}}e^{\beta (U\left(x^{*}\right)-U\left(x_{1}\right)-U\left(x_{2}\right))}\propto \frac{1}{|\lambda_{1}\left(x^{*}\right)|}e^{\beta (U\left(x^{*}\right)-U\left(x_{1}\right)-U\left(x_{2}\right))}, \end{array}$$

where *Z* is the normalizing constant of $e^{-\beta U(x)}$.

Next, we discuss how this estimate changes when skew matrices are applied. When the skew matrices are introduced, from lemma A.1 in [40], at the saddle point, there exists a unique negative real eigenvalue $\lambda_{1}^{\alpha }\left(x^{*}\right)<0$ for the perturbed Hessian matrix even if (I+αJ)H is not a symmetric matrix.

Then from Proposition 5 in [8], that negative eigenvalue of the perturbed Hessian is smaller than that of the un-perturbed Hessian matrix at the saddle point. This means that $\lambda_{1}^{\alpha }\left(x^{*}\right)\leq \lambda_{1}\left(x^{*}\right)<0$ holds.

Finally, from Theorem 5.1 in [41] and Theorem 2.12 in [21], this improvement of the negative eigenvalue of the saddle point directly leads to the larger Poincaré constant.

## Appendix E. Properties of a Skew-Symmetric Matrix

Here, we introduce the basic properties of the skew-symmetric matrices. Let us consider assume that d×d matrix $H^{\prime }=\left(I+\alpha J\right)H$ is diagonalizable. Then assume that matrix $H^{\prime }$ has *l* real eigenvalues $\lambda_{1},\ldots ,\lambda_{l}$ and 2m complex eigenvalues, $\mu_{1}=\alpha_{1}\pm i\beta_{1},\ldots ,\mu_{m}=\alpha_{m}\pm i\beta_{m}$. Thus, d=l+2m. We denote the corresponding eigenvectors as ${\left\{v_{j}\right\}}_{j=1}^{l}$ for real eigenvalues and ${\left\{w_{j}=a_{j}+ib_{j}\right\}}_{j=1}^{m}$ for complex eigenvalues ${\left\{\mu_{j}\right\}}_{j=1}^{m}$ and $\left\{{\bar{w}}_{j}\right\}$ for corresponding conjugate eigenvalues. Then, let us define a d×d matrix *V* as

(A39) $$\begin{array}{c} V=[v_{1},\ldots ,v_{l},a_{1},b_{1},\ldots ,a_{m},b_{m}]. \end{array}$$

Then, we can decompose $H^{\prime }$ into a block diagonal matrix [42];

(A40) $$\begin{array}{cc} \; & H^{\prime }V=VD \end{array}$$

(A41) $$D=\underset{:=A}{\underbrace{\left(\begin{array}{cccccccc} \lambda_{1} & \; & \\ \; & \ddots & \\ \; & \; & \lambda_{l}\\ \; & \; & & \alpha_{1} & 0\\ \; & \; & & 0 & \alpha_{1}\\ \; & \; & & \; & & \ddots \\ \; & \; & & \; & & \; & \alpha_{m} & 0\\ \; & \; & & \; & & \; & 0 & \alpha_{m} \end{array}\right)}}+\underset{:=B}{\underbrace{\left(\begin{array}{cccccccc} 0 & \; & \\ \; & \ddots & \\ \; & \; & 0\\ \; & \; & & 0 & \beta_{1}\\ \; & \; & & -\beta_{1} & 0\\ \; & \; & & \; & & \ddots \\ \; & \; & & \; & & \; & 0 & \beta_{m}\\ \; & \; & & \; & & \; & -\beta_{m} & 0 \end{array}\right)}}.$$

Thus, D:=A+B. Then, from the Taylor expansion and expressing its residual by integral, by defining $H\left(x\right):=\nabla^{2}U\left(x\right)$ we have

(A42) $$\begin{array}{cc} {\left(x-y\right)}^{⊤}\left(I+\alpha J\right)\left(\nabla U(x)-\nabla U(y)\right) & ={\left(x-y\right)}^{⊤}\left(\int_{0}^{1}\left(I+\alpha J\right)H\left(y+\tau \left(x-y\right)\right)\left(x-y\right)d\tau \right). \end{array}$$

Then, let us apply the Jordan canonical form here. If (I+αJ)H is diagonalizable, and it is decomposable by the Jordan canonical form shown in Equation (A40). Then, we can decompose (I+αJ)H as

(A43) $$\begin{array}{c} \left(I+\alpha J\right)H\left(x^{*}+\tau \left(x\left(t\right)-x^{*}\right)\right)=VDV^{-1}. \end{array}$$

Then, we obtain

(A44) $$\begin{array}{cc} {\left(x-y\right)}^{⊤}\left(I+\alpha J\right)\left(\nabla U(x)-\nabla U(y)\right) & ={\left(x-y\right)}^{⊤}\left(\int_{0}^{1}\left(I+\alpha J\right)H\left(y+\tau \left(x-y\right)\right)\left(x-y\right)d\tau \right)\\ \; & =\left(\int_{0}^{1}{\left(x-y\right)}^{⊤}V\left(A+B\right)V^{-1}\left(x-y\right)dt\right)\\ \; & =\left(\int_{0}^{1}{\left(x-y\right)}^{⊤}VAV^{-1}\left(x\left(t\right)-x^{*}\right)dt\right)\\ \; & \leq m\left(\alpha \right)∥x\left(t\right)-x^{*}{∥}^{2}. \end{array}$$

where m(α) is the constant that satisfies $m\left(\alpha \right)\leq \min\left\{\lambda_{1},\ldots ,\lambda_{l},\alpha_{1},\ldots ,\alpha_{m}\right\}$ for all *x*. Thus, the imaginary part never appears to the upper bound and we only need to focus on the largest real part of the eigenvalues, if the matrix is diagonalizable. Next subsection describes when the non-symmetric matrix $H^{\prime }$ is diagonalizable by focusing on the random matrix.

## Appendix F. Proof of Theorem 3

**Proof.** Since the potential function is *m*-strongly convex, the smallest eigenvalue of the Hessian matrix *H* is *m*, which is larger than 0. Thus, *H* and $H^{1/2}$ are regular matrices. With this in mind, we consider $H+H^{1/2}JH^{1/2}$ as a similar matrix of $H^{\prime }:=\left(I+J\right)H$. This is easily confirmed by

(A45) $$\begin{array}{c} H^{-1/2}\left(H+H^{1/2}JH^{1/2}\right)H^{1/2}=H^{\prime }. \end{array}$$

This means that to study the eigenvalues of $H^{\prime }$, we only need to study the similar matrix $A:=H+H^{1/2}JH^{1/2}$. By doing this, *A* is composed of symmetric and skew-symmetric matrices, which are easy to treat compared to $H^{\prime }$, where the term JH is difficult to analyze. For simplicity, we omit the dependency of *H* and $H^{\prime }$ on *x* in this section.

**Remark A1.** *Please note that we can eliminate the strong convexity of U, if H is a regular matrix. This means that H does not have* 0 *as an eigenvalue.*

For simplicity, we assume that the dimension *d* is an even number. We assume that the eigenvalues and eigenvectors of *A* are expressed as

(A46) $$\begin{array}{c} Aw_{j}=\mu_{j}w_{j}\Leftrightarrow A\left(a_{j}+ib_{j}\right)=\left(\alpha_{j}+i\beta_{j}\right)\left(a_{j}+ib_{j}\right). \end{array}$$

and $\alpha_{j}$ is ordered as $\alpha_{1}\leq \alpha_{2},\ldots$. In this section, we only consider the setting where all the eigenvalue and eigenvector are imaginary for notational simplicity. The extension to the general settings similar to Appendix E and the setting when is *d* is odd is straightforward.

We denote the eigenvalues and eigenvectors of *H* as ${\left\{\lambda_{j},v_{j}\right\}}_{j=1}^{d}$ and $v_{j}$s are linearly independent. In addition, we assume that $\lambda_{1}\leq ,\ldots ,\lambda_{d}$. From this definition, by checking the real parts and complex parts, the following relations are derived

(A47) $$\begin{array}{c} Aa_{j}=\alpha_{j}a_{j}-\beta b_{j}, \end{array}$$

(A48) $$\begin{array}{c} Ab_{j}=\alpha_{j}b_{j}+\beta a_{j}. \end{array}$$

thus, by the skew-symmetric property

(A49) $$\begin{array}{cc} a_{j}^{⊤}Aa_{j}+b_{j}^{⊤}Ab_{j} & =\alpha_{j}(∥a_{j}{∥}^{2}+∥b_{j}{∥}^{2})=\alpha_{j} \end{array}$$

(A50) $$\begin{array}{cc} \; & =a_{j}^{⊤}Ha_{j}+b_{j}^{⊤}Hb_{j}, \end{array}$$

and in the third equality, we used the property

(A51) $$\begin{array}{c} a_{j}^{⊤}H^{1/2}JH^{1/2}a_{j}=b_{j}^{⊤}H^{1/2}JH^{1/2}b_{j}=0, \end{array}$$

since $H^{1/2}JH^{1/2}$ is a skew-symmetric matrix. Then, we expand $a_{j}$ and $b_{j}$ by $v_{j}$ as

(A52) $$\begin{array}{c} a_{k}=\sum_{j=1}^{d}a_{k}^{⊤}v_{j} \end{array}$$

(A53) $$\begin{array}{c} b_{k}=\sum_{j=1}^{d}b_{k}^{⊤}v_{j}v_{j}, \end{array}$$

since $v_{j}$s are eigenvalues of *H*, which can be used as the basis for $R^{d}$. Then we substitute this into Equation (A50) and we have

(A54) $$\begin{array}{cc} \alpha_{k} & =\sum_{j=1}^{d}\lambda_{j}{\left(a_{k}^{⊤}v_{j}\right)}^{2}+\sum_{j=1}^{d}\lambda_{j}{\left(b_{k}^{⊤}v_{j}\right)}^{2}\geq \lambda_{1}\sum_{j=1}^{d}{\left(a_{k}^{⊤}v_{j}\right)}^{2}+{\left(b_{k}^{⊤}v_{j}\right)}^{2})=\lambda_{1}. \end{array}$$

This means that any real part of the eigenvalue of *A* is larger than $\lambda_{1}$ which is the smallest eigenvalue of *H*. Thus, if the $\alpha_{1}$ is the smallest real part of the eigenvalue of *A*, that is larger than the smallest eigenvalue of *H*. This concludes the proof.

In the same way,

(A55) $$\begin{array}{cc} \alpha_{k} & =\sum_{j=1}^{d}\lambda_{j}{\left(a_{k}^{⊤}v_{j}\right)}^{2}+\sum_{j=1}^{d}\lambda_{j}{\left(b_{k}^{⊤}v_{j}\right)}^{2}\leq \lambda_{d}\sum_{j=1}^{d}{\left(a_{k}^{⊤}v_{j}\right)}^{2}+{\left(b_{k}^{⊤}v_{j}\right)}^{2})=\lambda_{d}, \end{array}$$

which means any real part of the eigenvalues of *A* is smaller than the largest eigenvalue of *H*. Thus, if α is the largest real part of the eigenvalues of *A*, it is smaller than the largest eigenvalue of *H*.

Equality condition:

Next, we discuss when the equality holds for $\alpha_{1}=\lambda_{1}$. First, we assume that eigenvalues of *H* are distinct, thus, there is only one eigenvector for $\lambda_{1}$. Later, we discuss if eigenvalues are not distinct. From Equation (A54), we have

(A56) $$\begin{array}{cc} \alpha_{1} & =\sum_{j=1}^{d}\lambda_{j}{\left(a_{1}^{⊤}v_{j}\right)}^{2}+\sum_{j=1}^{d}\lambda_{j}{\left(b_{1}^{⊤}v_{j}\right)}^{2}\geq \lambda_{1}\sum_{j=1}^{d}{\left(a_{1}^{⊤}v_{j}\right)}^{2}+{\left(b_{1}^{⊤}v_{j}\right)}^{2})=\lambda_{1}, \end{array}$$

in general. Please note that if $a_{1}$ and $b_{1}$ does not correspond to $v_{1}$, then $\lambda_{j\neq 1}>\lambda_{1}$ must appear in the summation and equality never holds. So, the condition is

(A57) $$\begin{array}{cc} \; & a_{1},b_{1}\propto v_{1}, \end{array}$$

must hold for the equality.

Based on this, let us assume that $w_{1}=ca_{1}+ic^{\prime }b_{1}$ where $c^{2}+c^{{}^{\prime }2}=1$. We consider the case $a_{1}=b_{1}=v_{1}$. Then we need to solve the simultaneous equations

(A58) $$\begin{array}{cc} \; & A\left(ca_{1}+ic^{\prime }b_{1}\right)=\left(\lambda_{1}+i\beta_{1}\right)\left(ca_{1}+ic^{\prime }b_{1}\right)=\left(\lambda_{1}c-c^{\prime }\beta_{1}\right)v_{1}+i\left(c\beta_{1}+\lambda_{1}c^{\prime }\right)v_{1}, \end{array}$$

this is obtained by the definition of the eigenvalue of *A* and

(A59) $$\begin{array}{cc} \; & A\left(ca_{1}+ic^{\prime }b_{1}\right)=\lambda_{1}^{1/2}c\left(I\lambda_{1}^{1/2}+\alpha H^{1/2}J\right)v_{1}+i\lambda_{1}^{1/2}c^{\prime }\left(I\lambda_{1}^{1/2}+\alpha H^{1/2}J\right)v_{1}, \end{array}$$

this is obtained from the definition of eigenvalues of *H*. Then multiplying $v_{1}$ from the left, we obtain $c\beta_{1}=0$ and $c^{\prime }\beta_{1}=0$. Thus, $\beta_{1}=0$. $\beta_{1}=0$ means $b_{1}=0$ from the property of the complex eigenvectors. Thus, we obtain $w_{1}=a_{1}=v_{1}$ for $\lambda_{1}=\alpha_{1}$. Then, the following relation holds,

(A60) $$\begin{array}{c} \lambda_{1}v_{1}=Av_{1}=Hv_{1}+\alpha H^{1/2}JH^{1/2}v_{1}=\lambda_{1}v_{1}+\alpha \lambda_{1}^{1/2}H^{1/2}Jv_{1}. \end{array}$$

Since $\lambda_{1}\neq 0$ and $H^{1/2}$ has the inverse matrix, this condition indicates that

(A61) $$\begin{array}{c} \alpha Jv_{1}=0. \end{array}$$

This is the condition that $\lambda_{1}=\alpha_{1}$ holds. The same relation can be derived for $\lambda_{d}=\alpha_{d}$.

Next, we assume that eigenvalues of *H* are not distinct. Let us denote the set of eigenvectors of the eigenvalue $\lambda_{1}^{0}$ as $\left\{v_{1}^{0}\right\}$. Please note that if $a_{1}$ and $b_{1}$ does not included in $V_{1}^{0}$, then $\lambda_{j\neq 1}>\lambda_{1}$ must appear and equality never holds. Thus

(A62) $$\begin{array}{cc} \; & a_{1},b_{1}\in V_{1}^{0} \end{array}$$

must hold for equality. Based on this, let us assume that $w_{1}=ca_{1}+ic^{\prime }b_{1}$ where $c^{2}+c^{{}^{\prime }2}=1$. We consider the case $a_{1}\neq b_{1}$. Then

(A63) $$\begin{array}{cc} \; & H^{-1/2}A\left(ca_{1}+ic^{\prime }b_{1}\right)=\lambda_{1}^{-1/2}\left(\lambda_{1}+i\beta_{1}\right)\left(ca_{1}+ic^{\prime }b_{1}\right)\\ \; & H^{-1/2}\left(H+\alpha H^{1/2}JH^{1/2}\right)\left(ca_{1}+ic^{\prime }b_{1}\right)=\lambda_{1}^{1/2}c\left(I+\alpha J\right)a_{1}+i\lambda_{1}^{1/2}c^{\prime }\left(I+\alpha J\right)b_{1}, \end{array}$$

then we obtain the condition

(A64) $$\begin{array}{cc} \; & \lambda_{1}c\alpha Ja_{1}=-\beta_{1}c^{\prime }b_{1} \end{array}$$

(A65) $$\begin{array}{cc} \; & \lambda_{1}c^{\prime }\alpha Jb_{1}=\beta_{1}ca_{1}. \end{array}$$

□

## Appendix G. Proofs of Random Matrices

### Appendix G.1. Proof of Theorem 5

**Proof.** The proof is the straightforward consequence of lemma in [43], that is Lemma in ([43]) *If $f(x_{1},\ldots ,x_{m})$ is a polynomial in real variables $x_{1},\ldots ,x_{m}$, which is not identically zero, then the subset $N_{m}=\left\{\left(x_{1},\ldots ,x_{m}\right)|f\left(x_{1},\ldots ,x_{m}\right)=0\right\}$ of the Euclidean m-space $R^{m}$ has the Lebesgue measure zero.* We use this lemma to prove that the probability of $\lambda_{1}=\alpha_{1}$ is 0 by showing that the probability mass of $\lambda_{1}=\alpha_{1}$ has Lebesgue measure zero. We use the same notation as in Appendix F. Recall Equation (A64), which is the condition of equality about $\lambda_{1}=\alpha_{1}$. We express the elements of $a_{1}$ and $b_{1}$ as $a_{1}={\left(a_{1}^{1},\ldots ,a_{1}^{d}\right)}^{⊤}$ and $b_{1}={\left(b_{1}^{1},\ldots ,b_{1}^{d}\right)}^{⊤}$. Then the equality condition can be written as

(A66) $$\begin{array}{cc} \; & \sum_{i=1}^{d}\left(\sum_{j=1}^{d}\lambda_{1}c\alpha J_{i,j}a_{1}^{j}+\beta_{1}c^{\prime }b_{1}^{i}\right){)}^{2}+\sum_{i=1}^{d}\left(\sum_{j=1}^{d}\lambda_{1}c^{\prime }\alpha J_{i,j}b_{1}^{j}-\beta_{1}ca_{1}^{i}\right){)}^{2}=0. \end{array}$$

Then we define the polynomial about $\left\{J_{i.j}\right\}$

(A67) $$\begin{array}{c} f\left(J_{1,2},\ldots ,J_{d-1,d}\right)=\sum_{i=1}^{d}\left(\sum_{j=1}^{d}\lambda_{1}c\alpha J_{ij}a_{1}^{j}+\beta_{1}c^{\prime }b_{1}^{i}\right){)}^{2}+\sum_{i=1}^{d}\left(\sum_{j=1}^{d}\lambda_{1}c^{\prime }\alpha J_{ij}b_{1}^{j}-\beta_{1}ca_{1}^{i}\right){)}^{2}. \end{array}$$

To apply lemma of [43], we must confirm that $f(J_{1,2},\ldots ,J_{d-1,d})$ is not always 0. This is clear from the definition of *f* since we generate $J_{1,2},\ldots ,J_{d-1,d}$ randomly from the distribution that is absolutely continuous with respect to Lebesgue measure and $\lambda_{1}\neq 0$ and $c^{2}+c^{\prime 2}=1$ and either $a_{1},b_{1}\neq 0$. Then, given an evaluation point *x*, from lemma of [43], the subset of $\left\{J_{i,j}\right\}\in R^{d(d-1)/2}$ that satisfies $f(J_{1,2},\ldots ,J_{d-1,d})=0$ has Lebesque measure zero. Thus, if we generate $\left\{J_{i,j}\right\}$ from the probability measure which is absolutely continuous with respect to Lebesque measure, (such as Gaussian distribution), $f(J_{1,2},\ldots ,J_{d-1,d})=0$ holds probability 0. This concludes the proof. □

### Appendix G.2. Proof of Lemma 1

**Proof.** We first discuss the condition about $\mathrm{Ker}J_{0}=\left\{0\right\}$. Since $J=J_{0}⊗I_{d}$, and we denote the set of eigenvalues of $J_{0}$ as $\left\{\omega_{i}\right\}$. In general, the eigenvalues of the matrix that is composed of the Kronecker product with two matrices, e.g., *A* and *B*, are given as the product of each eigenvalue of *A* and *B* [44]. Thus, since *J* is the Kronecker product of $J_{0}$ and $I_{d}$, if $J_{0}$ does not have 0 as an eigenvalue, *J* does not have 0 as an eigenvalue. Next, we discuss another equality condition. We use the similar notation as in Appendix F, but now the dimension of the matrix *J* is dN. We express the eigenvalue which has the smallest real part as $\lambda_{1}^{\alpha }$ and its eigenvector as $\omega_{1}^{\alpha }=a_{1}+ib_{1}$. The elements of $a_{1}$ and $b_{1}$ as $a_{1}={\left(a_{1}^{1},\ldots ,a_{1}^{d},a_{1}^{d+1},\ldots ,a_{1}^{dN}\right)}^{⊤}\in R^{dN}$ and $b_{1}={\left(b_{1}^{1},\ldots ,b_{1}^{d},\ldots ,b_{1}^{d+1},\ldots ,b_{1}^{dN}\right)}^{⊤}$. We also express these as $a_{1}={\left(a_{1}^{\left(1\right)},\ldots ,a_{1}^{\left(N\right)}\right)}^{⊤}\in R^{dN}$ where $a_{1}^{\left(i\right)}={\left(a_{1}^{(i-1)d+1},\ldots ,a_{1}^{id}\right)}^{⊤}\in R^{d}$. We use the Kronecker product property:

(A68) $$\begin{array}{c} Ja_{1}=\left(J_{0}⊗I_{d}\right)a_{1}={\left(\sum_{i=1}^{N}J_{0|i,1}a_{1}^{\left(i\right)},\ldots ,\sum_{i=1}^{N}J_{0|i,N}a_{1}^{\left(i\right)}\right)}^{⊤}, \end{array}$$

where $J_{0|i,j}$ indicates the element of *i*-th row and *j*-th column of $J_{0}$ where we use the property of the Kronecker product and the Vec operator in the second equality [44]. The proof is almost similar to Appendix G.1. Then the equality condition can be written as

(A69) $$\begin{array}{cc} \; & \sum_{n=1}^{N}{∥\lambda_{1}c\alpha \sum_{i}J_{0|i,n}a_{1}^{\left(i\right)}+\beta_{1}c^{\prime }b_{1}^{\left(n\right)}∥}^{2}+\sum_{n=1}^{N}{∥\lambda_{1}c^{\prime }\alpha \sum_{i}J_{0|i,n}b_{1}^{\left(i\right)}+\beta_{1}ca_{1}^{\left(n\right)}∥}^{2}=0, \end{array}$$

where ∥·∥ is the *d*-dimensional Euclidean norm since $a_{1}^{\left(n\right)},b_{1}^{\left(n\right)}\in R^{d}$. Then we define the polynomial about $\left\{J_{i.j}\right\}$

(A70) $$\begin{array}{c} f\left(J_{1,2},\ldots ,J_{N-1,N}\right)=\sum_{n=1}^{N}{∥\lambda_{1}c\alpha \sum_{i}J_{0|i,n}a_{1}^{\left(i\right)}+\beta_{1}c^{\prime }b_{1}^{\left(n\right)}∥}^{2}+\sum_{n=1}^{N}{∥\lambda_{1}c^{\prime }\alpha \sum_{i}J_{0|i,n}b_{1}^{\left(i\right)}+\beta_{1}ca_{1}^{\left(n\right)}∥}^{2}. \end{array}$$

In a similar discussion with Appendix G.1, it is clear that *f* is not always 0. Thus, given an evaluation point *x*, from lemma of [43], the subset of $\left\{J_{i,j}\right\}\in R^{N(N-1)/2}$ that satisfies $f(J_{1,2},\ldots ,J_{N-1,d})=0$ has Lebesque measure zero. Thus, if we generate $\left\{J_{i,j}\right\}$ from the probability measure which is absolutely continuous with respect to Lebesque measure, (such as Gaussian distribution), $f(J_{1,2},\ldots ,J_{N-1,N})=0$ holds probability 0. This concludes the proof. □

### Appendix G.3. Extending the Theorem to the Path

About Theorem 5 and Lemma 1, the statement holds true when we fix an evaluation point *x*. To ensure the acceleration, we need to extend Theorem 5 and Lemma 1 from a single evaluation point to the path of the stochastic process for S-LD, S-PLD, S-ULD, and S-PULD.

First, the condition of $\mathrm{Ker}J_{0}=\left\{0\right\}$ is not related to the evaluation point. Thus, we need to consider the equality condition for Reλ1α=λ10. As for this condition, as we had seen in Theorem 5 and Lemma 1, if we generate the random matrix *J* which is absolutely continuous with respect to Lebesgue measure, then the equality condition is not satisfied with probability 1 at the given evaluation point. The important point in those proof is to prove that the event when the equality holds has Lebesgue measure 0 at the given evaluation point using the lemma of [43].

Let us consider when two evaluation points are given (e.g., $x_{1}$, $x_{2}$), and we check whether the random matrix *J* satisfies the above equality condition or not. We can easily prove that at each evaluation point, such an event (we express them as $S_{1}$ and $S_{2}$) has Lebesgue measure 0 using the lemma of [43] (We refer to this as $P(S_{1})=0$ and $P(S_{2})=0$ where *P* is the law induced by generating the random matrix that has independent d(d−1)/2 elements). So, the volume of the event of sum of $S_{1}$ and $S_{2}$ are also 0 ($P(S_{1}⋃S_{2}=0$). By repeating this procedure, when given a finite number of evaluation points, $\left(x_{1},\ldots ,x_{k}\right)$, the sum of such probability is 0 (this indicates $P(S_{1}⋃S_{2},\ldots ,⋃S_{k})=0$).

When we consider the discretized dynamics of S-LD, S-PLD, and so on, and update samples up to *k*-iterations, then there exist *k* evaluation points. So, by applying the above discussion, we can ensure that along the path of the discretized dynamics, the equality condition does not hold with probability 1. On the other hand, as for the continuous dynamics, the evaluation point is infinite, thus when we cannot conclude that the probability that the equality does not hold is 1.

## Appendix H. Proof of Theorem 6

We use the same notation as in Appendix F. We consider the expansion concerning α and we consider the following setting,

(A71) $$\begin{array}{cc} \; & w_{j}:=v_{j}+\delta v_{j} \end{array}$$

(A72) $$\begin{array}{cc} \; & \mu_{j}:=\lambda_{j}+\delta \lambda_{j}, \end{array}$$

which indicates that by introducing the skew-acceleration terms, the pairs of eigenvalues and eigenvectors of $H^{\prime }$ are expressed by the small perturbation for the eigenvalues and eigenvectors of *H*. Since ${\left\{v_{j}\right\}}_{j=1}^{d}$ are the eigenvalues of *H* and they can be used as an orthogonal basis, thus we expand δv by this basis. We obtain

(A73) $$\begin{array}{c} \delta v_{j}=\sum_{k\neq j}^{d}c_{jk}v_{k}, \end{array}$$

where $c_{jk}=\delta v_{j}^{⊤}v_{k}$.

### Appendix H.1. Asymptotic Expansion When the Smallest Eigenvalue of H(x) Is Positive

We work on the similar matrix of $H^{\prime }$, that is H+αV where $V:=H^{1/2}JH^{1/2}$. See Appendix G.1 for the detail. Please note that this similar matrix only exists when the smallest eigenvalue of H(x) is positive. Thus, the following discussion cannot apply to the case at the saddle point, where negative eigenvalues appear. We discuss the saddle point expansion later.

From the definition, we have

(A74) $$\begin{array}{c} H^{\prime }w_{j}=Hw_{j}+\alpha Vw_{j}=\mu_{j}w_{j}=\left(\lambda_{j}+\delta \lambda_{j}\right)\left(v_{j}+\delta v_{j}\right), \end{array}$$

We rearrange this equation as

(A75) $$\begin{array}{c} Hv_{j}+H\delta v_{j}+\alpha Vv_{j}+\alpha V\delta v_{j}=\lambda_{j}v_{j}+\delta \lambda_{j}v_{j}+\lambda_{j}\delta v_{j}+\delta \lambda_{j}\delta v_{j}. \end{array}$$

First, we focus on the first-order expansion. This means we neglect high-order terms. Then, we have

(A76) $$\begin{array}{c} Hv_{j}+H\delta v_{j}+\alpha Vv_{j}=\lambda_{j}v_{j}+\delta \lambda_{j}v_{j}+\lambda_{j}\delta v_{j}. \end{array}$$

By multiplying $v_{j}$ to Equation (A76) from the left-hand side, we have

(A77) $$\begin{array}{c} \lambda_{j}+\lambda_{j}v_{j}^{⊤}\delta v_{j}+\alpha v_{j}^{⊤}Vv_{j}=\lambda_{j}+\delta \lambda_{j}+\lambda_{j}v_{j}^{⊤}\delta v_{j}, \end{array}$$

Since $v_{j}^{⊤}Vv_{j}=0$ due to the skew-symmetric property of *V*. Thus, we have

(A78) $$\begin{array}{c} \delta \lambda_{j}=0, \end{array}$$

up to the first-order expansion. Then we substitute this into Equation (A76) and multiplying $v_{i}$ where i≠j, we have

(A79) $$\begin{array}{c} \lambda_{i}c_{ji}+\alpha v_{i}^{⊤}Vv_{j}=\lambda_{j}c_{ji}. \end{array}$$

Then we have

(A80) $$\begin{array}{c} c_{ji}=\frac{\alpha v_{i}^{⊤}Vv_{j}}{\lambda_{j}-\lambda_{i}}. \end{array}$$

Then we obtain

(A81) $$\begin{array}{c} \delta v_{j}=\alpha \sum_{i\neq j}^{d}\frac{v_{i}^{⊤}Vv_{j}}{\lambda_{j}-\lambda_{i}}v_{i}. \end{array}$$

We substitute this into Equation (A75), and multiplying $v_{j}^{⊤}$, we have

(A82) $$\begin{array}{cc} \; & v_{j}^{⊤}H\alpha \sum_{i\neq j}^{d}\frac{v_{i}^{⊤}Vv_{j}}{\lambda_{j}-\lambda_{i}}v_{i}+\alpha v_{j}^{⊤}Vv_{j}+\alpha v_{j}^{⊤}V\alpha \sum_{i\neq j}^{d}\frac{v_{i}^{⊤}Vv_{j}}{\lambda_{j}-\lambda_{i}}v_{i}\\ \; & =\delta \lambda_{j}v_{j}^{⊤}v_{j}+\lambda_{j}v_{j}^{⊤}\alpha \sum_{i\neq j}^{d}\frac{v_{i}^{⊤}Vv_{j}}{\lambda_{j}-\lambda_{i}}v_{i}+\delta \lambda_{j}v_{j}^{⊤}\alpha \sum_{i\neq j}^{d}\frac{v_{i}^{⊤}Vv_{j}}{\lambda_{j}-\lambda_{i}}v_{i}. \end{array}$$

Since $v_{j}^{⊤}Vv_{j}=0$ and $v_{j}^{⊤}v_{i}=0$ and $v_{j}^{⊤}v_{j}=1$, we have

(A83) $$\begin{array}{c} \alpha^{2}\sum_{i\neq j}^{d}\frac{v_{i}^{⊤}Vv_{j}}{\lambda_{j}-\lambda_{i}}v_{j}^{⊤}Vv_{i}=\delta \lambda_{j}. \end{array}$$

Thus, we have

(A84) $$\begin{array}{c} \mu_{j}-\lambda_{j}=\alpha_{j}+i\beta_{j}-\lambda_{j}=-\alpha^{2}\sum_{i\neq j}^{d}\frac{{\left(v_{i}^{⊤}Vv_{j}\right)}^{2}}{\lambda_{j}-\lambda_{i}}. \end{array}$$

Thus, by taking the real part, and note that Reλj(α)=αj, we have

(A85) Reλj(α)−λj=α2Re∑i≠jd(vi⊤Vvj)2λi−λj+O(α3)=α2∑i≠jdλiλj(vi⊤Jvj)2λi−λj+O(α3).

This concludes the proof.

### Appendix H.2. Expansion of the Eigenvalue at the Saddle Point

Here we derive the formula of the expansion of the eigenvalue at the saddle point. Since the smallest eigenvalue is negative, we cannot use the similar matrix as shown above. Instead, we use the relation,

(A86) $$\begin{array}{c} \mu_{j}Hw_{j}=H\mu_{j}w_{j}=H\left(I+\alpha J\right)Hw_{j} \end{array}$$

where we used the definition of the eigenvalues and eigenvectors. Here, we express $H^{\prime }:=\left(I+\alpha J\right)H$ and its pairs of eigenvalues and eigenvectors as ${\left\{\left(\mu_{i},w_{i}\right)\right\}}_{i=1}^{d}$. As introduced in the above, we substitute the expansion to Equation (A86), then we obtain

(A87) $$\begin{array}{c} \left(\lambda_{j}+\delta \lambda_{j}\right)H\left(v_{j}+\delta v_{j}\right)=H\left(I+\alpha J\right)H\left(v_{j}+\delta v_{j}\right) \end{array}$$

Then, in the same way as above, since ${\left\{v_{j}\right\}}_{j=1}^{d}$ are the eigenvalues of *H* and they can be used as an orthogonal basis, we expand δv by this basis. This means

(A88) $$\begin{array}{c} \delta v_{j}=\sum_{k=1}^{d}c_{jk}v_{k}, \end{array}$$

where $c_{jk}=\delta v_{j}^{⊤}v_{k}$. By multiplying $v_{i}$ to Equation (A87) where i≠j from left-hand side and neglecting high-order terms, we have

(A89) $$\begin{array}{c} c_{ji}=\frac{\lambda_{j}}{\lambda_{j}-\lambda_{i}}\left(v_{i}^{⊤}\alpha Jv_{i}\right). \end{array}$$

Next, Then by multiplying $v_{j}$ to Equation (A87) from left-hand side, we have

(A90) $$\begin{array}{c} v_{j}H\left(\alpha J\right)H\delta v_{j}=\left(\delta \lambda_{j}\right)\left(\lambda_{j}+\lambda_{j}v_{j}^{⊤}\delta v_{j}\right) \end{array}$$

Then by substituting $\delta v_{j}$ with coefficient Equation (A89), we have

(A91) $$\begin{array}{cc} \delta \lambda_{j}\; & =\alpha^{2}\sum_{i\neq j}^{d}\frac{\lambda_{i}\lambda_{j}{\left(v_{i}^{⊤}Jv_{j}\right)}^{2}}{\lambda_{i}-\lambda_{j}}+O\left(\alpha^{3}\right) \end{array}$$

This concludes the proof.

## Appendix I. Convergence Rate of Parallel Sampling Schemes

### Appendix I.1. Proof of Lemma 2

First, we introduce the notations. We express the random variables of S-PLD as $Y_{t}^{⊗N}$. We express the measure induced by S-PLD as $\mu_{t}^{⊗N}\left(\alpha \right)$, which uses the αJ as an interaction term. Thus, we express the measure of PLD as $\mu_{kh}^{⊗N}\left(0\right)$, we can decompose the measure as marginals. We also denote the marginal measure of S-PLD for $Y_{t}^{\left(n\right)}$$\nu_{t}^{\left(n\right)}\left(\alpha \right)$. Please note that initial distribution is $\mu_{0}^{⊗N}$ and its marginals are $\mu_{0}$ as defined in Assumption 4.

Please note that the marginal measure of PLD is the same as those of LD if the initial measures are all the same, thus each marginal satisfy the Poincaré constant $m_{0}$. This is also the result of the tensorization property of the spectral gap (Proposition 4.3.1 in Bakry et al. [19]).

As for the initial condition, from the fact that $\chi^{2}$ divergence is the special case of Renyi divergence (α=4), and from the tensorization property of the Renyi divergence (see Theorem 28 in [45]), we have

(A92) $$\begin{array}{c} \chi^{2}\left(\mu_{t}^{⊗N}\left(0\right),\pi^{⊗N}\right)\leq e^{-2\beta^{-1}m_{0}t}\chi^{2}\left(\mu_{0}^{⊗N},\pi^{⊗N}\right)=\sum_{n=1}^{N}e^{-2\beta^{-1}m_{0}t}\chi^{2}\left(\mu_{0},\pi \right). \end{array}$$

Then we have

(A93) $$\begin{array}{c} \chi^{2}\left(\mu_{t}^{⊗N}\left(0\right),\pi^{⊗N}\right)\leq e^{-2\beta^{-1}m_{0}t}\chi^{2}\left(\mu_{0}^{⊗N},\pi^{⊗N}\right)=Ne^{-2\beta^{-1}m_{0}t}\chi^{2}\left(\mu_{0},\pi \right). \end{array}$$

If the skew acceleration is applied, from the same discussion as S-LD (see Appendix C.1), S-PLD has the Poincaré constant which is larger than $m_{0}$. We express it as $m\left(\alpha ,N\right)\left(\geq m_{0}\right)$. Then we have

(A94) $$\begin{array}{c} \chi^{2}\left(\mu_{t}^{⊗N}\left(\alpha \right),\pi^{⊗N}\right)\leq Ne^{-2\beta^{-1}m\left(\alpha ,N\right)t}\chi^{2}\left(\mu_{0},\pi \right). \end{array}$$

At first, since there exists a constant *N* in the convergence bound, this bound seems not useful. However, as we discussed below, when we bound the bias or variance, these bound is meaningful. For example, let us consider approximating the true expectation ∫f(x)dπ(x) by the ensemble samples $\frac{1}{N}\sum_{n=1}^{N}f\left(X_{t}^{\left(n\right)}\right)$. Then we are interested in bounding the error

(A95) $$\begin{array}{c} \left|E\frac{1}{N}\sum_{n=1}^{N}f\left(X_{k}^{\left(n\right)}\right)-\int_{R^{d}}fd\pi \right|. \end{array}$$

For this purpose, we can bound this by 2-Wasserstein distance as

(A96) $$\begin{array}{c} \left|E\frac{1}{N}\sum_{n=1}^{N}f\left(X_{k}^{\left(n\right)}\right)-\int_{R^{d}}fd\pi \right|,\leq \frac{L_{f}}{\sqrt{N}}W_{2}\left(\mu_{kh}^{⊗N}\left(\alpha \right),\pi^{⊗N}\right) \end{array}$$

where we assumed that *f* shows $L_{f}$ lipschitzness and used the fact that $\frac{1}{N}\sum_{n=1}^{N}f\left(x^{\left(n\right)}\right)$ shows $L_{f}/\sqrt{N}$ lipschitzness.

To bound the distance, we use the basic relation

(A97) $$\begin{array}{c} W_{2}^{2}\left(\nu_{kh}\left(\alpha \right),\pi^{⊗N}\right)\leq 2\frac{1}{m(\alpha ,N)}\chi^{2}\left(\mu_{kh}^{⊗N}\left(\alpha \right),\pi^{⊗N}\right), \end{array}$$

where m(α,N) is the Poincaré constant. This is established by the definition of Wasserstein distance and $\chi^{2}$-divergence, see [46] for the detail. Then combined with above relations, we obtain the bias bound of S-PLD as

(A98) $$\begin{array}{c} \left|E\frac{1}{N}\sum_{n=1}^{N}f\left(X_{k}^{\left(n\right)}\right)-\int_{R^{d}}fd\pi \right|\leq L_{f}\sqrt{\frac{2}{m(\alpha ,N)}}e^{-\beta^{-1}m\left(\alpha ,N\right)kh}\chi^{2}{\left(\mu_{0},\pi \right)}^{1/2}. \end{array}$$

In the same way, we obtain the bias bound of PLD as

(A99) $$\begin{array}{c} \left|E\frac{1}{N}\sum_{n=1}^{N}f\left(X_{k}^{\left(n\right)}\right)-\int_{R^{d}}fd\pi \right|\leq L_{f}\sqrt{\frac{2}{m_{0}}}e^{-\beta^{-1}m_{0}kh}\chi^{2}{\left(\nu_{0},\pi \right)}^{1/2}. \end{array}$$

Thus, while the explicit dependency on *N* disappeared, but S-PLD shows faster convergence through the relation of $m\left(\alpha ,N\right)\geq m_{0}$. Moreover, if we use the skew matrices, which does not satisfy the equality condition, we have $m\left(\alpha ,N\right)>m_{0}$.

### Appendix I.2. Proof for S-ULD

We can characterize the convergence rate almost in the same way as Appendix C.2. The derivation is the same above, thus we only show the result

(A100) $$\begin{array}{c} \left|E\frac{1}{N}\sum_{n=1}^{N}f\left(X_{k}^{\left(n\right)}\right)-\int_{R^{d}}fd\pi \right|\leq L_{f}\sqrt{\frac{2}{m(\alpha ,N)}}\sqrt{\frac{1+\bar{\epsilon }}{1-\bar{\epsilon }}}e^{-\lambda_{\gamma }/2kh}\chi^{2}{\left(\nu_{0}^{0},\pi \right)}^{1/2}. \end{array}$$

where $\bar{\epsilon }$ and $\lambda_{\gamma }$ is given as follows.

(A101) $$\begin{array}{c} \lambda_{\gamma }=\frac{\Lambda (\gamma ,\bar{\epsilon }\min\left(\gamma ,\gamma^{-1}\right))}{1+\bar{\epsilon }\min\left(\gamma ,\gamma^{-1}\right)}, \end{array}$$

and

(A102) $$\begin{array}{cc} \; & \Lambda \left(\gamma ,\epsilon \right)=\frac{\gamma \Sigma^{-1}-\frac{1}{1+\frac{m_{0}\Sigma^{-1}}{\beta }}}{2}-\frac{1}{2}\sqrt{{\left(S_{--}-S_{++}\right)}^{2}+{\left(S_{-+}\right)}^{2}}, \end{array}$$

(A103) $$\begin{array}{cc} \; & S_{--}=\epsilon \lambda_{ham}, \end{array}$$

(A104) $$\begin{array}{cc} \; & S_{-+}=-\epsilon \left(R_{ham}+\gamma \Sigma^{-1}/2\right), \end{array}$$

(A105) $$\begin{array}{cc} \; & S_{++}=\gamma \Sigma^{-1}-\epsilon , \end{array}$$

(A106) $$\begin{array}{cc} \; & \lambda_{ham}=1-{\left(1+\frac{m\left(\alpha ,N\right)\Sigma^{-1}}{\beta }\right)}^{-1}, \end{array}$$

(A107) $$\begin{array}{cc} \; & \epsilon =\bar{\epsilon }\min\left(\gamma ,\gamma^{-1}\right), \end{array}$$

where $\bar{\epsilon }$ is arbitrary sufficiently small positive value such that $\Lambda (\gamma ,\bar{\epsilon }\min\left(\gamma ,\gamma^{-1}\right))>0$ is satisfies. and

(A108) $$\begin{array}{c} R_{ham}\leq \sqrt{\max\{M,2\}}. \end{array}$$

## Appendix J. Proof of Theorem 7

We show our theorem again with explicit constants

**Theorem** **A3.**
*Under Assumptions 1–7, for any k∈N and any $h\in (0,1\wedge \frac{m}{4M^{2}})$ obeying kh≥1 and βm≥2, we have*

(A109) $$\begin{array}{cc} \; & \left|E\frac{1}{N}\sum_{n=1}^{N}f\left(X_{k}^{\left(n\right)}\right)-\int_{R^{d}}fd\pi \right|\\ \; & \leq L_{f}\sqrt{{\tilde{C}}_{0}^{2}\sqrt{\delta }+{\tilde{C}}_{1}^{2}\sqrt{h}}k\eta +L_{f}\sqrt{\frac{2}{m(\alpha ,N)}}\chi^{2}{\left(\mu_{0},\pi \right)}^{1/2}e^{-\beta^{-1}m\left(\alpha ,N\right)kh}. \end{array}$$

*where*

(A110) $$\begin{array}{cc} \; & {\tilde{C}}_{0}^{2}=\left(12+8\left(\kappa_{0}+2b+\frac{2d}{\beta }\right)\right)\left(\beta C_{0}+\sqrt{\beta C_{0}}\right), \end{array}$$

(A111) $$\begin{array}{cc} \; & {\tilde{C}}_{1}^{2}=\left(12+8\left(\kappa_{0}+2b+\frac{2d}{\beta }\right)\right)\left(C_{1}+\sqrt{C_{1}}\right) \end{array}$$

(A112) $$\begin{array}{cc} \; & C_{0}={\left(1+\alpha \right)}^{2}\left(M^{2}\left(\kappa_{0}+2\left(1\vee \frac{1}{m}\right)\left(b+2{\left(1+\alpha \right)}^{2}B^{2}+\frac{d}{\beta }\right)\right)+B^{2}\right), \end{array}$$

(A113) $$\begin{array}{cc} \; & C_{1}=6\left(1+\alpha^{2}\right)M^{2}\left(\beta C_{0}+d\right), \end{array}$$

Then obtained bound is $O(kh\cdot h^{1/4})$, which is independent of *N*. Thus, this result is much better than those in [18]. Additionally, note that we can derive the similar bias bound for skew-SGHMC in the same way as skew-SGLD.

**Proof.** For notational simplicity, we express the random variables of skew-SGLD which uses the αJ as an interaction term as $X_{k}^{⊗N}$ and those of S-PLD as $Y_{k}^{⊗N}$. In this section, for simplicity, we express them as $X_{k}$ and $Y_{k}$. We denote the measure of $X_{k}$ and $Y_{k}$ as $\nu_{kh}^{⊗N}$ and $\mu_{kh}^{⊗N}$. We also denote the marginal measure of $X_{k}^{\left(n\right)}$ and $Y_{k}^{\left(n\right)}$ as $\mu_{kh}^{\left(n\right)}$ and $\nu_{kh}^{\left(n\right)}$. Then, we first decompose the bias as

(A114) $$\begin{array}{cc} \; & \left|E\frac{1}{N}\sum_{n=1}^{N}f\left(X_{k}^{\left(n\right)}\right)-\int_{R^{d}}fd\pi \right|\\ \; & =\left|E\frac{\sum_{n=1}^{N}f\left(X_{k}^{\left(n\right)}\right)}{N}-E\frac{\sum_{n=1}^{N}f\left(Y_{k}^{\left(n\right)}\right)}{N}+E\frac{\sum_{n=1}^{N}f\left(Y_{k}^{\left(n\right)}\right)}{N}-\int_{R^{d}}fd\pi \right|\\ \; & \leq \left|E\frac{1}{N}\sum_{n=1}^{N}f\left(X_{k}^{\left(n\right)}\right)-E\frac{1}{N}\sum_{n=1}^{N}f\left(Y_{k}^{\left(n\right)}\right)\right|+\left|E\frac{1}{N}\sum_{n=1}^{N}f\left(Y_{k}^{\left(n\right)}\right)-\int_{R^{d}}fd\pi \right|\\ \; & \leq \frac{L_{f}}{N}\sum_{i=1}^{N}W_{2}\left(\nu_{kh}^{\left(n\right)}\left(\alpha \right),\mu_{kh}^{\left(n\right)}\left(\alpha \right)\right)+\frac{L_{f}}{\sqrt{N}}\underset{\left(i\right)}{\underbrace{W_{2}\left(\mu_{kh}^{{}^{⊗}N}\left(\alpha \right),\pi^{⊗N}\right)}}, \end{array}$$

where we used the Jensen inequality for the first term in the last inequality and we move $\frac{1}{N}\sum_{i=1}^{N}$ outside the |·|. In addition, each expectation only depends on the marginal measures $\mu^{\left(i\right)}$ in the first term and we use the property of the 2-Wasserstein (2-W) distance. Furthermore, we decompose the first term as

(A115) $$\begin{array}{c} \frac{L_{f}}{N}\sum_{n=1}^{N}W_{2}\left(\mu_{kh}^{\left(n\right)}\left(\alpha \right),\nu_{kh}^{\left(n\right)}\left(\alpha \right)\right)\;\leq \frac{L_{f}}{N}\left(\sum_{n=1}^{N}\underset{\left(ii\right)}{\underbrace{W_{2}\left(\nu_{kh}^{\left(n\right)}\left(\alpha \right),\mu_{kh}^{\left(n\right)}\left(0\right)\right)}}+\underset{\left(iii\right)}{\underbrace{W_{2}\left(\mu_{kh}^{\left(n\right)}\left(\alpha \right),\mu_{kh}^{\left(n\right)}\left(0\right)\right)}}\right), \end{array}$$

where $\mu_{kh}^{\left(n\right)}\left(0\right)$ denotes the measure induced by PLD, which is the naive parallel sampling without a skew-symmetric interaction. In conclusion, our task is to bound each (i), (ii), (iii) terms in the above. Bounding (i) is already discussed in Appendix I.1. Next, we work on (ii) and (iii). Following [10], we use weighted CKP inequality to bound the 2-W distance. From Bolley and Villani [47], using the weighted CKP inequality, we can bound each 2-W distance by the relative entropy (KL divergence). This weighted CKP inequality indicates that

(A116) $$\begin{array}{c} W_{2}\left(\nu_{kh}^{\left(n\right)}\left(\alpha \right),\mu_{kh}^{\left(n\right)}\left(0\right)\right)\leq C_{\mu_{kh}^{\left(n\right)}\left(0\right)}\left(\mathrm{KL}{\left(\nu_{kh}^{\left(n\right)}\left(\alpha \right)|\mu_{kh}^{\left(n\right)}\left(0\right)\right)}^{1/2}+{\left(\frac{\mathrm{KL}(\nu_{kh}^{\left(n\right)}\left(\alpha \right)|\mu_{kh}^{\left(n\right)}\left(0\right))}{2}\right)}^{1/4}\right), \end{array}$$

with

(A117) $$\begin{array}{c} C_{\mu_{kh}^{\left(n\right)}\left(0\right)}=2\underset{\lambda >0}{\inf}{\left(\frac{1}{\lambda }\left(\frac{3}{2}+\log\int_{R^{d}}e^{\lambda ∥x^{\left(n\right)}{∥}^{2}}d\mu_{kh}^{\left(n\right)}\left(0\right)\right)\right)}^{1/2}. \end{array}$$

and

(A118) $$\begin{array}{c} W_{2}\left(\mu_{kh}^{\left(n\right)}\left(\alpha \right),\mu_{kh}^{\left(n\right)}\left(0\right)\right)\leq C_{\mu_{kh}^{\left(n\right)}\left(0\right)}\left(\mathrm{KL}{\left(\mu_{kh}^{\left(n\right)}\left(\alpha \right)|\mu_{kh}^{\left(n\right)}\left(0\right)\right)}^{1/2}+{\left(\frac{\mathrm{KL}(\mu_{kh}^{\left(n\right)}\left(\alpha \right)|\mu_{kh}^{\left(n\right)}\left(0\right))}{2}\right)}^{1/4}\right), \end{array}$$

with

(A119) $$\begin{array}{c} C_{\mu_{kh}^{\left(n\right)}\left(0\right)}=2\underset{\lambda >0}{\inf}{\left(\frac{1}{\lambda }\left(\frac{3}{2}+\log\int_{R^{d}}e^{\lambda ∥x^{\left(n\right)}{∥}^{2}}d\mu_{kh}^{\left(n\right)}\left(0\right)\right)\right)}^{1/2}. \end{array}$$

We point out that using $C_{\mu_{kh}^{\left(i\right)}\left(0\right)}$ not $C_{\nu_{kh}^{\left(i\right)}\left(\alpha \right)}$ and $C_{\mu_{kh}^{\left(i\right)}\left(\alpha \right)}$ in weighted CKP inequality is important. This is because since $\mu_{kh}^{\left(i\right)}\left(0\right)$ is the constant based on the parallel-chain Monte Carlo without skew-symmetric term, thus the parallel chain can be decomposed each independent chains. Thus, $C_{\mu_{kh}^{\left(i\right)}}$ actually does not depend on *i* and it does not depend on *N* and shows O(d) dependency. However, $C_{\nu_{kh}^{\left(i\right)}\left(\alpha \right)}$ and $C_{\mu_{kh}^{\left(i\right)}\left(\alpha \right)}$ show O(dN) which shows linear dependency on *N* since there is an interaction term between parallel chains and we cannot decompose the parallel chain easily. Thus, this results in unsatisfactory dependency on *N*. This is the reason we introduced $\mu_{kh}^{\left(i\right)}\left(0\right)$ in our theoretical analysis. Please note that since $\mu_{kh}^{\left(n\right)}\left(0\right)$ is induced by the naive parallel chain, each marginal is independent with each other and takes the same measure if the initial measure is the same. Thus, $\mu_{kh}^{\left(1\right)}\left(0\right)=\cdots =\mu_{kh}^{\left(N\right)}\left(0\right)$. From now on, we express the marginal as $\mu_{kh}\left(0\right)$ for simplicity. Thus, $C_{\mu_{kh}^{\left(1\right)}\left(0\right)}=\cdots =C_{\mu_{kh}^{\left(N\right)}\left(0\right)}=C_{\mu_{kh}\left(0\right)}$. Then substituting the above WKP inequalities and using the Jensen inequality, we obtain

(A120) $$\begin{array}{cc} \; & \left|E\frac{1}{N}\sum_{n=1}^{N}f\left(X_{k}^{\left(n\right)}\right)-E\frac{1}{N}\sum_{n=1}^{N}f\left(Y_{k}^{\left(n\right)}\right)\right|\\ \; & \leq L_{f}C_{\mu_{kh}\left(0\right)}\frac{1}{N}\sum_{n=1}^{N}\left(\mathrm{KL}{\left(\nu_{kh}^{\left(n\right)}\left(\alpha \right)|\mu_{kh}\left(0\right)\right)}^{1/2}+{\left(\frac{\mathrm{KL}(\nu_{kh}^{\left(n\right)}\left(\alpha \right)|\mu_{kh}\left(0\right))}{2}\right)}^{1/4}\right.\\ \; & \left.\;\;\;\;\;\;\;\;+\mathrm{KL}{\left(\mu_{kh}^{\left(n\right)}\left(\alpha \right)|\mu_{kh}\left(0\right)\right)}^{1/2}+{\left(\frac{\mathrm{KL}(\mu_{kh}^{\left(n\right)}\left(\alpha \right)|\mu_{kh}\left(0\right))}{2}\right)}^{1/4}\right)\\ \; & \leq L_{f}C_{\mu_{kh}\left(0\right)}\left(\;{\left(\sum_{n=1}^{N}\frac{\mathrm{KL}(\nu_{kh}^{\left(n\right)}\left(\alpha \right)|\mu_{kh}\left(0\right))}{N}\;\right)}^{\frac{1}{2}}\;+\;{\left(\;\sum_{n=1}^{N}\frac{\mathrm{KL}(\nu_{kh}^{\left(n\right)}\left(\alpha \right)|\mu_{kh}\left(0\right))}{2N}\;\right)}^{\frac{1}{4}}\;\right.\\ \; & \left.\;\;\;\;\;\;+\;{\left(\;\sum_{n=1}^{N}\frac{\mathrm{KL}(\mu_{kh}^{\left(n\right)}\left(\alpha \right)|\mu_{kh}\left(0\right))}{N}\;\right)}^{\frac{1}{2}}\;+\;{\left(\;\sum_{n=1}^{N}\frac{\mathrm{KL}(\mu_{kh}^{\left(n\right)}\left(\alpha \right)|\mu_{kh}\left(0\right))}{2N}\;\right)}^{\frac{1}{4}}\;\right)\;. \end{array}$$

To analyze the discretization error, we use the following key lemma: **Lemma A1.** 
*Assume that there exist random variables ${\left\{X_{i}\in \Omega_{i}\right\}}_{i=1}^{N}$ and ${\left\{Y_{i}\in \Omega_{i}\right\}}_{i=1}^{N}$. We denote the product space as $\Omega^{⊗N}:=\Omega_{1}\times \ldots \Omega_{N}$. Let us introduce $X=\left(X_{1},\ldots ,X_{N}\right)\in \Omega^{⊗N}$ and $Y=\left(Y_{1},\ldots ,Y_{N}\right)\in \Omega^{⊗N}$. Let us express their joint probability measures as expressed as $P\left(X\right):=P\left(X_{1},\ldots ,X_{N}\right)$, $Q\left(Y\right):=Q\left(Y_{1},\ldots ,Y_{N}\right)$, let us denote the marginal measures of each Xs and Ys as ${\left\{P_{i}\left(X_{i}\right)\right\}}_{i=1}^{N}$ and ${\left\{Q_{i}\left(Y_{i}\right)\right\}}_{i=1}^{N}$. If $P_{i}<<Q_{i}$ holds, we have*

(A121) $$\begin{array}{c} \sum_{i=1}^{N}\mathrm{KL}\left(P_{i}\left(X_{i}\right)∥Q_{i}\left(Y_{i}\right)\right)\leq \mathrm{KL}\left(P\left(X\right)∥Q\left(Y\right)\right), \end{array}$$

A proof is given in Appendix J.1. We apply this lemma as

(A122) $$\begin{array}{cc} \; & \sum_{n=1}^{N}\mathrm{KL}\left(\mu_{kh}^{\left(n\right)}|\mu_{kh}\left(0\right)\right)\leq \mathrm{KL}\left(\nu_{kh}^{⊗N}|\mu_{kh}^{⊗N}\left(0\right)\right), \end{array}$$

(A123) $$\begin{array}{cc} \; & \sum_{n=1}^{N}\mathrm{KL}\left(\mu_{kh}^{\left(n\right)}\left(\alpha \right)|\mu_{kh}\left(0\right)\right)\leq \mathrm{KL}\left(\mu_{kh}^{⊗N}\left(\alpha \right)\right)|\mu_{kh}^{⊗N}\left(0\right))). \end{array}$$

Combining these results with the above bias bound, we obtain

(A124) $$\begin{array}{cc} \; & \left|E\frac{1}{N}\sum_{n=1}^{N}f\left(X_{k}^{\left(n\right)}\right)-E\frac{1}{N}\sum_{n=1}^{N}f\left(Y_{k}^{\left(n\right)}\right)\right|\\ \; & \leq L_{f}C_{\mu_{kh}\left(0\right)}\left({\left(\frac{\mathrm{KL}(\nu_{kh}^{⊗N}\left(\alpha \right)|\mu_{kh}^{⊗N}\left(0\right))}{N}\;\right)}^{\frac{1}{2}}\;+{\left(\frac{\mathrm{KL}(\nu_{kh}^{⊗N}\left(\alpha \right)|\mu_{kh}^{⊗N}\left(0\right))}{2N}\;\right)}^{\frac{1}{4}}\;\right.\\ \; & \left.\;\;\;\;\;\;+{\left(\frac{\mathrm{KL}(\mu_{kh}^{⊗N}\left(\alpha \right)\left(\alpha \right)|\mu_{kh}^{⊗N}\left(0\right))}{N}\;\right)}^{\frac{1}{2}}\;+{\left(\frac{\mathrm{KL}(\mu_{kh}^{⊗N}\left(\alpha \right)|\mu_{kh}^{⊗N}\left(0\right))}{2N}\;\right)}^{\frac{1}{4}}\;\right)\;.\; \end{array}$$

Thus, we need to bound $\mathrm{KL}(\mu_{kh}^{\left(i\right)}\left(\alpha \right)|\mu_{kh}^{⊗N}\left(0\right))$ and $\mathrm{KL}(\nu_{kh}^{⊗N}\left(\alpha \right)|\mu_{kh}^{⊗N}\left(0\right))$ and $C_{\mu_{kh}\left(0\right)}$. We can upper-bound them using the results of [2]. For that purpose, we need to replace the constants in [2] as we show in the below. Here, we discuss how the constants in the assumption are changed in the ensemble scheme. We define

(A125) $$\begin{array}{c} \nabla u^{⊗N}\left(x^{⊗N}\right):=\left(\nabla u\left(x^{\left(1\right)}\right),\ldots ,\nabla u\left(x^{\left(N\right)}\right)\right) \end{array}$$

First, we focus on the smoothness condition. From Assumption 2 and lemma 8 in [18], we have

(A126) $$\begin{array}{c} ∥\left(I+\alpha J\right)\nabla u^{⊗N}\left(x^{⊗N},z\right)-\left(I+\alpha J\right)\nabla u^{⊗N}\left(y^{⊗N},z\right))∥\leq M\left(1+\alpha \right)∥x^{⊗N}-y^{⊗N}∥. \end{array}$$

where the norm in the right-hand side is the Euclidean norm in $R^{dN}$. Next, we discuss the smoothness condition. Define $\nabla U_{\alpha }\left(x^{⊗N}\right):=\nabla U^{⊗N}\left(x^{⊗N}\right)+\alpha J\nabla U^{⊗N}\left(x^{⊗N}\right)$. Then, Let $x^{⊗N}\in R^{dN}$ and under the assumptions 1 to 6, we have

(A127) $$\begin{array}{c} x^{⊗N}\cdot \nabla U_{\alpha }\left(x^{⊗N}\right)\geq m{∥x^{⊗N}∥}^{2}-bN. \end{array}$$

Next, we check about the condition of the drift function at the origin: ∥∇u(0,z)∥≤B. We can calculate in the same way as the smoothness condition. Then we have

(A128) $$\begin{array}{c} ∥\left(I+\alpha J\right)\nabla U^{⊗N}\left(0^{⊗N}\right)∥\leq B\sqrt{N}\left(1+\alpha \right). \end{array}$$

Next, we study the condition about the stochastic gradient: $E[∥\nabla \hat{U}\left(x\right)-\nabla U\left(x\right){∥}^{2}]\leq 2\delta \left(M^{2}{∥x∥}^{2}+B^{2}\right)$. This can be easily modified to

(A129) $$\begin{array}{cc} \; & E[∥\left(I+\alpha J\right)\nabla {\hat{U}}^{⊗N}\left(x^{⊗N}\right)-\left(I+\alpha J\right)\nabla U^{⊗N}\left(x^{⊗N}\right){∥}^{2}]\\ \; & \leq {\left(1+\alpha \right)}^{2}E\left[\nabla {\hat{U}}^{⊗N}\left(x^{⊗N}\right)-\nabla U^{⊗N}\left(x^{⊗N}\right){∥}^{2}\right]\\ \; & \leq {\left(1+\alpha \right)}^{2}\sum_{i=1}^{N}E\left[\nabla \hat{U}\left(x^{\left(i\right)}\right)-\nabla U\left(x^{\left(i\right)}\right){∥}^{2}\right]\\ \; & \leq {\left(1+\alpha \right)}^{2}\sum_{i=1}^{N}2\delta \left(M^{2}{∥x^{\left(i\right)}∥}^{2}+B^{2}\right)\\ \; & \leq 2\delta {\left(1+\alpha \right)}^{2}\left(M^{2}{∥x^{⊗N}∥}^{2}+NB^{2}\right). \end{array}$$

Finally, we discuss about the initial condition: $\kappa_{0}:=\log\int_{R^{d}}e^{{∥x∥}^{2}}p_{0}\left(x\right)dx<\infty$. We assume that the initial probability distribution is $\mu_{0}^{⊗N}\left(X_{0}^{⊗N}\right)=\mu_{0}\left(X_{0}^{\left(1\right)}\right)\times \ldots \times \mu_{0}\left(X_{0}^{\left(N\right)}\right)$, which means that all the marginal probability is the same. Then

(A130) $$\begin{array}{cc} \kappa_{0}^{⊗N} & :=\log\int_{R^{dN}}e^{∥x^{⊗N}{∥}^{2}}\mu_{0}^{⊗N}\left(x^{⊗N}\right)dx^{⊗N}=\log\prod_{n=1}^{N}\left(\int_{R^{d}}e^{∥x^{\left(n\right)}{∥}^{2}}\mu_{0}\left(x^{\left(n\right)}\right)dx\right)=N\kappa_{0}. \end{array}$$

In this way, the constants in the assumptions are modified and expressed with *N* and α. Then combined with the results of [2], we can derive the following relations

(A131) $$\begin{array}{cc} \; & C_{\nu_{kh}\left(0\right)}\leq 12+8\left(\kappa_{0}+2b+\frac{2d}{\beta }\right), \end{array}$$

(A132) $$\begin{array}{cc} \; & \mathrm{KL}\left(\nu_{kh}^{⊗N}|\mu_{kh}^{⊗N}\left(0\right)\right)\leq N\left(C_{0}\beta \delta +C_{1}\eta \right)k\eta , \end{array}$$

(A133) $$\begin{array}{cc} \; & \mathrm{KL}\left(\mu_{kh}^{⊗N}\left(\alpha \right)|\mu_{kh}^{⊗N}\left(0\right)\right)\leq N\frac{\beta }{2}\alpha^{2}M^{2}\left(\kappa_{0}+\frac{b+d/\beta }{m}\right)k\eta , \end{array}$$

where

(A134) $$\begin{array}{cc} \; & C_{0}={\left(1+\alpha \right)}^{2}\left(M^{2}\left(\kappa_{0}+2\left(1\vee \frac{1}{m}\right)\left(b+2{\left(1+\alpha \right)}^{2}B^{2}+\frac{d}{\beta }\right)\right)+B^{2}\right), \end{array}$$

(A135) $$\begin{array}{cc} \; & C_{1}=6\left(1+\alpha^{2}\right)M^{2}\left(\beta C_{0}+d\right). \end{array}$$

This concludes the proof. □

### Appendix J.1. Proof of Lemma A1

**Proof.** We prove this lemma using the Donsker–Varadhan representation of the relative entropy [48]. The relative entropy admits the dual representation as:

(A136) $$\begin{array}{c} \mathrm{KL}\left(P\left(X\right)∥Q\left(Y\right)\right)=\underset{T:\Omega^{⊗N}\to R}{\sup}E_{P(X)}\left[T\right]-\log E_{Q(Y)}\left[e^{T}\right], \end{array}$$

where supremum is taken over all function *T* of which the expectation of $e^{T}$ and *T* are finite. We then restrict the function class into a class $F\left(T\right)=\{T\left(X\right)|\exists T_{i}:\Omega_{i}\to R,s.t.T\left(X\right)=\sum_{i=1}^{N}T_{i}\left(X_{i}\right)\}$ where each expectation of $e^{T_{i}}$ and $T_{i}$ are finite. Then by definition,

(A137) $$\begin{array}{c} \mathrm{KL}\left(P\left(X\right)∥Q\left(Y\right)\right)=\underset{T:\Omega \to R}{\sup}E_{P(X)}\left[T\right]-\log E_{Q(Y)}\left[e^{T}\right]\geq \underset{T\in F}{\sup}E_{P(X)}\left[\sum_{i}T_{i}\right]-\log E_{Q(Y)}\left[e^{\sum_{i}T_{i}}\right]. \end{array}$$

Then we have

(A138) $$\begin{array}{cc} \mathrm{KL}(P(X)∥Q(Y)) & \geq \underset{T\in F}{\sup}\sum_{i}E_{P_{i}\left(X_{i}\right)}\left[T_{i}\right]-\log\prod_{i}E_{Q_{i}\left(Y_{i}\right)}\left[e^{T_{i}}\right]\\ \; & =\underset{T\in F}{\sup}\sum_{i}\left(E_{P_{i}\left(X_{i}\right)}\left[T_{i}\right]-\log E_{Q_{i}\left(Y_{i}\right)}\left[e^{T_{i}}\right]\right)\\ \; & =\sum_{i}\underset{T_{i}:\Omega_{i}\to R}{\sup}E_{P_{i}\left(X_{i}\right)}\left[T_{i}\right]-\log E_{Q_{i}\left(Y_{i}\right)}\left[e^{T_{i}}\right]\\ \; & =\sum_{i=1}^{N}\mathrm{KL}\left(P_{i}\left(X_{i}\right)∥Q_{i}\left(Y_{i}\right)\right). \end{array}$$

□

## Appendix K. Order Expansion

### Bias Expansion for S-PLD

Recall that the bias of S-PLD is

(A139) $$\begin{array}{cc} \; & \left|E\frac{1}{N}\sum_{n=1}^{N}f\left(X_{k}^{\left(n\right)}\right)-\int_{R^{d}}fd\pi \right|\\ & \leq L_{f}\sqrt{{\tilde{C}}_{0}^{2}\sqrt{\delta }+{\tilde{C}}_{1}^{2}\sqrt{h}}k\eta +L_{f}\sqrt{\frac{2}{m(\alpha ,N)}}\chi^{2}\left(\mu_{0}\right){,\pi )}^{1/2}e^{-\beta^{-1}m\left(\alpha ,N\right)kh}. \end{array}$$

where

(A140) $$\begin{array}{cc} \; & {\tilde{C}}_{0}^{2}=\left(12+8\left(\kappa_{0}+2b+\frac{2d}{\beta }\right)\right)\left(\beta C_{0}+\sqrt{\beta C_{0}}\right), \end{array}$$

(A141) $$\begin{array}{cc} \; & {\tilde{C}}_{1}^{2}=\left(12+8\left(\kappa_{0}+2b+\frac{2d}{\beta }\right)\right)\left(C_{1}+\sqrt{C_{1}}\right) \end{array}$$

(A142) $$\begin{array}{cc} \; & C_{0}={\left(1+\alpha \right)}^{2}\left(M^{2}\left(\kappa_{0}+2\left(1\vee \frac{1}{m}\right)\left(b+2{\left(1+\alpha \right)}^{2}B^{2}+\frac{d}{\beta }\right)\right)+B^{2}\right), \end{array}$$

(A143) $$\begin{array}{cc} \; & C_{1}=6\left(1+\alpha^{2}\right)M^{2}\left(\beta C_{0}+d\right), \end{array}$$

First, we discuss the convergence of the continuous dynamics. Using the eigenvalue expansion in Theorem 6, with some positive constant $d_{0}$, we have

(A144) $$\begin{array}{c} m\left(\alpha ,N\right)\approx m_{0}+\alpha^{2}d_{0}+O\left(\alpha^{3}\right). \end{array}$$

Then by assuming $\alpha^{2}$ is small enough and considering the Tayler expansion, we have

(A145) $$\begin{array}{cc} \; & L_{f}\sqrt{\frac{2}{m(\alpha ,N)}}\chi^{2}{\left(\mu_{0},\pi \right)}^{1/2}e^{-\beta^{-1}m\left(\alpha ,N\right)t}\approx L_{f}\chi^{2}{\left(\mu_{0},\pi \right)}^{1/2}\sqrt{2}\left(\frac{1}{\sqrt{m_{0}}}-\frac{d_{0}}{2m_{0}^{3/2}}\alpha^{2}\right)e^{-\beta^{-1}m_{0}t}. \end{array}$$

As for the discretization and stochastic gradient error, using the Taylor expansion, there exists a positive constant $d_{1}$ and $d_{2}$, such that

(A146) $$\begin{array}{c} L_{f}\sqrt{{\tilde{C}}_{0}^{2}\sqrt{\delta }+{\tilde{C}}_{1}^{2}\sqrt{h}}k\eta \approx \left(d_{1}\alpha +d_{2}\alpha^{2}+\mathrm{Const}\right)kh. \end{array}$$

Combining these terms, we have

(A147) $$\begin{array}{cc} \; & \left|E\frac{1}{N}\sum_{n=1}^{N}f\left(X_{k}^{\left(n\right)}\right)-\int_{R^{d}}fd\pi \right|\leq \left(d_{1}\alpha +d_{2}\alpha^{2}\right)kh-\alpha^{2}L_{f}\chi^{2}{\left(\mu_{0},\pi \right)}^{1/2}\frac{1}{\sqrt{2}m_{0}^{3/2}}e^{-\beta^{-1}m_{0}t}+\mathrm{Const}. \end{array}$$

Thus, there exists an optimal $\alpha^{*}$, which minimizes the bias. Please note that at k=0, acceleration always occurs. As *k* goes to infinity, the second third terms 0, thus the first term will be dominant, which means we have larger discretization and stochastic gradient error.

## Appendix L. Hyperparameters of the Proposed Algorithm

Here we discuss how to set hyperparameters in the algorithm. There are three hyperparameters, $\alpha_{0}$, η, and *c*. We numerically found that setting c=0.95 work well for real dataset including LDA experiment, and Bayesian neural network regression and classification. For toy dataset, we set c=0.9.

As for $\alpha_{0}$ and η, we empirically found that using the following scaling trick works well for real dataset including LDA experiment, and Bayesian neural network regression and classification,

(A148) $$\begin{array}{c} \alpha_{0}\approx \frac{1}{\sqrt{\frac{1}{N^{2}}\sum_{n}\nabla U{\left(x_{0}^{\left(n\right)}\right)}^{2}}}Nh. \end{array}$$

and using $\eta \approx 0.1\alpha_{0}$. The intuition is that the magnitude of the gradient can be very different in each dimension, so we introduce the scaling by the gradient. We also multiply *h* so that the stochastic gradient and discretization error of the skew term will not be dominant compared to usual gradient term. Finally, we multiply some constant so that $\alpha_{0}$ will not be too small.

## Appendix M. Proof of Theorem 8

In this section, we derive the upper-bound of the bias of skew-SGLD based on [23]. This approach requires us to use the logarithmic Sobolev inequality [19], which is stronger than the Poincaré inequality. First, we present the definition of the logarithmic Sobolev inequality. We say that π on $R^{d}$ with L satisfies the logarithmic Sobolev inequality with constant λ in case for all function *f* on $R^{d}$ with $\int_{R^{d}}u^{2}d\pi =1$,

(A149) $$\begin{array}{c} \int_{R^{d}}f^{2}\ln f^{2}d\pi \leq \frac{2}{\lambda }\int_{R^{d}}-fLfd\pi . \end{array}$$

This logarithmic Sobolev inequality is stronger than the Poincaré inequality and induces the convergence in KL divergence. See [19] for details. It was proved in [2,18] that our dynamics, LD, SLD, PLD, S-PLD, and skew-SGLD satisfy the logarithmic Sobolev inequalities under our assumptions. We express the constant of the logarithmic Sobolev inequality for skew-SGLD as λ(α,N). This constant depends on the skew matrices and the Poincaré constant. We estimate this constant in Appendix M.1.

To upper-bound the bias, here we control the KL divergence. We denote the law of skew-SGLD at iteration *k* with interaction strength α as $\mu_{kh}^{⊗N}\left(\alpha \right)$. We upper-bound the bias by 2-Wasserstein distance

(A150) $$\begin{array}{c} \left|E\frac{1}{N}\sum_{n=1}^{N}f\left(X_{k}^{\left(n\right)}\right)-\int_{R^{d}}fd\pi \right|,\leq \frac{L_{f}}{\sqrt{N}}W_{2}\left(\mu_{kh}^{⊗N}\left(\alpha \right),\pi^{⊗N}\right). \end{array}$$

Then, from the transportation inequality [19],

(A151) $$\begin{array}{c} W_{2}\left(\mu_{k},\pi \right)\leq \sqrt{\frac{2}{\lambda (\alpha ,N)}\mathrm{KL}\left(\mu_{kh}^{⊗N}\left(\alpha \right)|\pi^{⊗N}\right)}. \end{array}$$

Thus, we will upper bound the KL divergence using the technique in [23]. However, in the original proof, a full gradient ∇U is used so we replace it with the stochastic gradient. Moreover, we introduce the skew interaction term.

First, Lemma 11 in [23] is modified to

(A152) $$\begin{array}{c} E_{\pi^{⊗}N}{∥\nabla U^{⊗N}∥}^{2}\leq \frac{dNM}{\beta }. \end{array}$$

Then Lemma 12 in [23] is modified to

(A153) $$\begin{array}{c} E_{\mu }{∥\nabla U^{⊗N}∥}^{2}\leq 4M^{2}\lambda \mathrm{KL}\left(\mu |\pi^{⊗N}\right)+\frac{2dNM}{\beta }, \end{array}$$

for any integrable μ.

Herein after, we drop ⊗N from $X^{⊗N}$, $\nabla U^{⊗N}$, and $\nabla {\hat{U}}^{⊗N}$ for notational simplicity. We focus on skew-SGLD at iteration *k*, we consider the following SDE for t∈(kh,(k+1)h]

(A154) $$\begin{array}{c} dX_{t}=-\left(I+\alpha J\right)\nabla \tilde{U}\left(X_{k}\right)dt+\sqrt{2\beta^{-1}}dw_{t}, \end{array}$$

where $\nabla \tilde{U}\left(X_{k}\right)$ is the stochastic gradient conditioned on $X_{k}$. The solution of this SDE is

(A155) $$\begin{array}{c} X_{\left(k+1\right)}=X_{k}-\left(I+\alpha J\right)\nabla \tilde{U}\left(X_{k}\right)h+\sqrt{2\beta^{-1}}\epsilon . \end{array}$$

We would like to derive the continuity equation correspond to Equation (A154). Following [23], we express $X_{t}$ as $x_{t}$ and $X_{k}$ as $x_{0}$ for simplicity. Let $\rho_{0t}\left(x_{0},x_{t}\right)$ denote the joint distribution of $\left(x_{0},x_{t}\right)$. Then, the conditional and marginal relations are written as

(A156) $$\begin{array}{c} \rho_{0t}\left(x_{0},x_{t}\right)=\rho_{0}\left(x_{0}\right)\rho_{t|0}\left(x_{t}|x_{0}\right)=\rho_{t}\left(x_{t}\right)\rho_{0|t}\left(x_{0}|x_{t}\right). \end{array}$$

The conditional density $\rho_{t|0}\left(x_{t}|x_{0}\right)$ follows the FP equation

(A157) $$\begin{array}{cc} \; & \frac{\partial \rho_{t|0}\left(x_{t}|x_{0}\right)}{\partial t}=\nabla \cdot \left(\rho_{t|0}\left(x_{t}|x_{0}\right)\left(I+\alpha J\right)\nabla \tilde{U}\left(x_{0}\right)\right)+\beta^{-1}\Delta \rho_{t|0}\left(x_{t}|x_{0}\right), \end{array}$$

Then following [23], to derive the evolution of $\rho_{t}$, we take the expectation over $\rho_{0}\left(x_{0}\right)$

(A158) $$\begin{array}{cc} \frac{\partial \rho_{t}\left(x\right)}{\partial t}\; & =\int_{R^{d}}\frac{\partial \rho_{t|0}\left(x_{t}|x_{0}\right)}{\partial t}\rho_{0}\left(x_{0}\right)dx_{0}\\ \; & =\nabla \cdot \left(\rho_{t}\left(x_{t}\right)E_{\rho_{0|t}}\left[\left(I+\alpha J\right)\nabla \tilde{U}\left(x_{0}\right)|x_{t}=x\right]\right)+\beta^{-1}\Delta \rho_{t}\left(x\right). \end{array}$$

Then, we take the expectation regarding for the stochastic gradient in the above equation and include it into $E_{\rho_{0|t}}$ for notational simplicity. Then following the discussion of Lemma 3 in [23], we obtain

(A159) $$\begin{array}{cc} \frac{\partial \mathrm{KL}(\mu_{t}|\pi )}{\partial t} & \leq -\frac{3}{4}I\left(\mu_{t}^{⊗N}|\pi^{⊗N}\right)+2E_{\rho_{0t}}[∥\nabla U\left(X_{t}\right)-\nabla U\left(X_{0}\right){∥}^{2}]\\ \; & +2{\left(1+\alpha \right)}^{2}E_{\rho_{0t}}[∥\nabla U\left(X_{0}\right)-\nabla \tilde{U}\left(X_{0}\right){∥}^{2}]+2\alpha^{2}E_{\rho_{0t}}\left[\nabla U\left(x_{0}\right){∥}^{2}\right], \end{array}$$

where t∈(kh,(k+1)h] and

(A160) $$\begin{array}{c} X_{t}=X_{k}-t\left(I+\alpha J\right)\nabla U\left(X_{k}\right)+\sqrt{2t\beta^{-1}}\epsilon . \end{array}$$

Then, from [18], we can upper-bound the second term by

(A161) $$\begin{array}{cc} \; & E_{\rho_{0t}}[∥\nabla U\left(X_{0}\right)-\nabla \tilde{U}\left(X_{0}\right){∥}^{2}]\leq NC_{0}^{\prime }\delta , \end{array}$$

(A162) $$\begin{array}{cc} \; & C_{0}^{\prime }:=2\left(M^{2}\left(\kappa_{0}+2\left(1\vee \frac{1}{m}\right)\left(b+2{\left(1+\alpha \right)}^{2}B^{2}+\frac{d}{\beta }\right)\right)+B^{2}\right) \end{array}$$

and the third term is upper-bounded by

(A163) $$\begin{array}{cc} E_{\rho_{0t}}[∥\nabla U\left(X_{0}\right)-\nabla E_{\rho_{0t}}\left[\nabla U\left(x_{0}\right){∥}^{2}\right] & \leq 2M^{2}{∥x_{0}∥}^{2}+2NB^{2}\\ \; & \leq NC_{0}^{\prime }, \end{array}$$

where we used lemma 2 and 7 in [2]. Finally, from the original proof of [23] we obtain

(A164) $$\begin{array}{c} 2E_{\rho_{0t}}[∥\nabla U\left(X_{t}\right)-\nabla U\left(X_{0}\right){∥}^{2}]\leq 8t^{2}M^{4}\lambda \mathrm{KL}\left(\mu_{k}^{⊗N}|\pi^{⊗N}\right)+\frac{4t^{2}dNM^{3}}{\beta }+\frac{4tdNM^{2}}{\beta }. \end{array}$$

Then, in conclusion, under $h\in (0,1\wedge \frac{m}{4M^{2}})$ obeying kh≥1 and βm≥2, we obtain

(A165) $$\begin{array}{cc} \frac{d}{dt}\mathrm{KL}\left(\mu_{t}^{⊗N}|\pi^{⊗N}\right)\leq -\frac{3}{4}I\left(\mu_{t}^{⊗N}|\pi^{⊗N}\right) & +8t^{2}M^{4}\lambda \left(\alpha ,N\right)\mathrm{KL}\left(\mu_{k}^{⊗N}|\pi^{⊗N}\right)\\ \; & +\frac{4t^{2}dNM^{3}}{\beta }+\frac{4tdNM^{2}}{\beta }+2NC_{0}^{\prime }\left(\delta {\left(1+\alpha \right)}^{2}+\alpha^{2}\right). \end{array}$$

For simplicity, we assume that $h\in (0,\frac{m}{4M^{2}})$ and $\frac{m}{4M^{2}}<1$, then we obtain

(A166) $$\begin{array}{cc} \frac{d}{dt}\mathrm{KL}\left(\mu_{t}^{⊗N}|\pi^{⊗N}\right)\leq -\frac{3}{4}I\left(\mu_{t}^{⊗N}|\pi^{⊗N}\right)+ & 8t^{2}M^{4}\lambda \left(\alpha ,N\right)\mathrm{KL}\left(\mu_{k}^{⊗N}|\pi^{⊗N}\right)\\ \; & +\frac{t^{2}dNM}{\beta }\left(m+4M\right)+2NC_{0}^{\prime }\left(\delta {\left(1+\alpha \right)}^{2}+\alpha^{2}\right). \end{array}$$

Then using t∈(kh,(k+1)h], we obtain

(A167) $$\begin{array}{cc} \mathrm{KL}(\mu_{k+1}^{⊗N}|\pi^{⊗N})\leq & e^{-\frac{3}{2}\lambda \left(\alpha ,N\right)h}\left(1+16h^{3}M^{4}\lambda \right)\mathrm{KL}\left(\mu_{k}^{⊗N}|\pi^{⊗N}\right)\\ \; & +e^{-\frac{3}{2}\lambda \left(\alpha ,N\right)h}\left(\frac{2hdNM}{\beta }\left(m+4M\right)+8hNC_{0}^{\prime }\left(\delta {\left(1+\alpha \right)}^{2}+\alpha^{2}\right)\right). \end{array}$$

If $h\in (0,\frac{\lambda (\alpha ,N)}{4\sqrt{2}M^{2}})$, we obtain

(A168) $$\begin{array}{cc} \; & \mathrm{KL}\left(\mu_{k+1}^{⊗N}|\pi^{⊗N}\right)\leq e^{-\lambda (\alpha ,N)h}\mathrm{KL}\left(\mu_{k}^{⊗N}|\pi^{⊗N}\right)+\frac{2h^{2}dNM}{\beta }\left(m+4M\right)+8hNC_{0}^{\prime }\left(\delta {\left(1+\alpha \right)}^{2}+\alpha^{2}\right). \end{array}$$

From this one step inequality, we obtain

(A169) $$\begin{array}{cc} \; & \mathrm{KL}(\mu_{k}^{⊗N}|\pi^{⊗N})\\ \; & \leq e^{-\lambda (\alpha ,N)kh}\mathrm{KL}\left(\mu_{0}^{⊗N}|\pi^{⊗N}\right)+\frac{1}{1-e^{-\lambda (\alpha ,N)h}}\left(\frac{2h^{2}dNM}{\beta }\left(m+4M\right)+8hNC_{0}^{\prime }\left(\delta {\left(1+\alpha \right)}^{2}+\alpha^{2}\right)\right)\\ \; & \leq e^{-\lambda (\alpha ,N)kh}\mathrm{KL}\left(\mu_{0}^{⊗N}|\pi^{⊗N}\right)+\frac{2N}{\lambda (\alpha ,N)}\left(\frac{hdM}{\beta }\left(m+4M\right)+4C_{0}^{\prime }\left(\delta {\left(1+\alpha \right)}^{2}+\alpha^{2}\right)\right). \end{array}$$

Then, finally we obtain

(A170) $$\begin{array}{cc} \; & \left|E\frac{1}{N}\sum_{n=1}^{N}f\left(X_{k}^{\left(n\right)}\right)-\int_{R^{d}}fd\pi \right|\\ \; & \leq \frac{L_{f}}{\sqrt{N}}\sqrt{\frac{2}{\lambda (\alpha ,N)}\mathrm{KL}\left(\mu_{kh}^{⊗N}\left(\alpha \right)|\pi^{⊗N}\right)}\\ \; & \leq L_{f}\sqrt{\frac{2}{\lambda (\alpha ,N)}}\sqrt{e^{-\lambda (\alpha ,N)kh}\mathrm{KL}\left(\mu_{0}|\pi \right)+\frac{2}{\lambda (\alpha ,N)}\left(\frac{hdM}{\beta }\left(m+4M\right)+4C_{0}^{\prime }\left(\delta {\left(1+\alpha \right)}^{2}+\alpha^{2}\right)\right)}\\ \; & \leq L_{f}\sqrt{\frac{2}{\lambda (\alpha ,N)}}\sqrt{e^{-\lambda (\alpha ,N)kh}\mathrm{KL}\left(\mu_{0}|\pi \right)+\frac{C_{3}\left(\alpha \right)}{\lambda (\alpha ,N)}}, \end{array}$$

where

(A171) $$\begin{array}{cc} \; & C_{3}\left(\alpha \right):=2\frac{hdM}{\beta }\left(m+4M\right)+8C_{0}^{\prime }\left(\delta {\left(1+\alpha \right)}^{2}+\alpha^{2}\right), \end{array}$$

(A172) $$\begin{array}{cc} \; & C_{0}^{\prime }:=2\left(M^{2}\left(\kappa_{0}+2\left(1\vee \frac{1}{m}\right)\left(b+2{\left(1+\alpha \right)}^{2}B^{2}+\frac{d}{\beta }\right)\right)+B^{2}\right). \end{array}$$

Moreover, from Appendix M.1, the logarithmic Sobolev constant is

(A173) $$\begin{array}{c} \lambda \left(\alpha ,N\right):=\left(\frac{1}{{(1+\beta m\left(\alpha ,N\right)}^{-1}|C\left(m_{0}\right)\left|\right)2\pi e^{2}}+\frac{3}{2m(\alpha ,N)}\right), \end{array}$$

where

(A174) $$\begin{array}{c} -C\left(m_{0}\right):=E_{\pi^{⊗N}}[∥\nabla U^{⊗N}\left(x\right){∥]}^{1/2}+\sqrt{\frac{8}{m_{0}}E_{\pi^{⊗N}}[∥\nabla U^{⊗N}\left(x\right){{∥}^{2}]}^{1/2}}. \end{array}$$

### Appendix M.1. Estimation of the Logarithmic Sobolev Constant

In this section, we estimate the logarithmic Sobolev constants using the technique of restricted logarithmic Sobolev inequality, which was introduced in [49].

The technique of [49] estimates the constant of the logarithmic Sobolev inequality as follows. Assume that π on $R^{d}$ with L satisfies the Poincaré inequality with constant *m*. Then, for any function *u* on $R^{d}$ that satisfies

(A175) $$\begin{array}{c} \int_{R^{d}}ud\pi =0\;\mathrm{and}\;\int_{R^{d}}u^{2}d\pi =1, \end{array}$$

we find a constant *b* that satisfies

(A176) $$\begin{array}{c} \int_{R^{d}}u^{2}\ln u^{2}d\pi \leq b\int_{R^{d}}-uLud\pi . \end{array}$$

Then the logarithmic constant is larger than $2{\left(b+\frac{3}{m}\right)}^{-1}$. Thus, we only need to focus on the restricted function class to estimate a constant *b*. We slightly change the Lemma 3.2 of [49] that estimate the constant *b* in Equation (A176) to apply it in our setting. In Lemma 3.2 of [49], it was proved that if *u* on $R^{d}$ satisfies the conditions in Equation (A175), then for any t∈(0,1), we have

(A177) $$\begin{array}{cc} \; & \int_{R^{d}}-uLud\pi -t\pi e^{2}\int_{R^{d}}u^{2}\ln u^{2}d\pi \geq \left(1-t\right)m+t\beta \int_{R^{d}}\left(-\frac{1}{2}LU\left(x\right)-\pi e^{2}U\left(x\right)\right)u^{2}d\pi , \end{array}$$

where we assume that $\pi \propto e^{-\beta U(x)}$ satisfies the Poincaré inequality with constant *m*. If there exists a constant *C* such that

(A178) $$\begin{array}{c} -C\geq \beta \int_{R^{d}}\left(-\frac{1}{2}LU\left(x\right)-\pi e^{2}U\left(x\right)\right)u^{2}d\pi >-\infty , \end{array}$$

then by setting t=m/(m+|C|), we can show that

(A179) $$\begin{array}{cc} \; & \int_{R^{d}}-uLud\pi -m/\left(m+|C|\right)\pi e^{2}\int_{R^{d}}u^{2}\ln u^{2}d\pi >0. \end{array}$$

Thus, the constant *b* in Equation (A176) is b=t=m/(m+|C|) and the logarithmic constant is $2(m/(m+|C|)+\frac{3}{m}{)}^{-1}$.

Thus, We analyze the constant *C*. The first term of the integral in Equation (A178) is lower-bounded bounded by

(A180) $$\begin{array}{c} -E_{\pi }\left[LU\left(x\right)u^{2}\right]\geq -|E_{\pi }\left[U\left(x\right)LU\left(x\right)\right]{|}^{1/2}|E_{\pi }\left[u^{2}Lu^{2}\right]{|}^{1/2}\geq -2E_{\pi }[∥\nabla U\left(x\right){{∥}^{2}]}^{1/2}, \end{array}$$

where we used the property of L, see [19] for details. As for the second term, it is lower-bounded by

(A181) $$\begin{array}{cc} -|E_{\pi }\left[U\left(x\right)u^{2}\right]\geq -\sqrt{|E_{\pi }\left[U^{2}\left(x\right)u^{2}\right]} & \geq -\sqrt{\frac{1}{m}|E_{\pi }\left[(U\left(x\right)|u|)L(U\left(x\right)|u|)]\right|}\\ \; & \geq -\sqrt{\frac{8}{m}E_{\pi }{[∥\nabla U\left(x\right)∥}^{2}{]}^{1/2}}. \end{array}$$

Thus, by setting

(A182) $$\begin{array}{c} -C:=E_{\pi }{\left[∥\nabla U\left(x\right)∥\right]}^{1/2}+\sqrt{\frac{8}{m_{0}}E_{\pi }{[∥\nabla U\left(x\right)∥}^{2}{]}^{1/2}}, \end{array}$$

we can estimate the logarithmic constant as $2(m/(m+|C|)+\frac{3}{m}{)}^{-1}$.

In our setting, this is modified to

(A183) $$\begin{array}{c} \lambda \left(\alpha ,N\right)={\left(\frac{1}{{(1+\beta m\left(\alpha ,N\right)}^{-1}|C\left(m_{0}\right)\left|\right)2\pi e^{2}}+\frac{3}{2m(\alpha ,N)}\right)}^{-1}. \end{array}$$

where

(A184) $$\begin{array}{c} -C\left(m_{0}\right):=E_{\pi^{⊗N}}[∥\nabla U^{⊗N}\left(x\right){∥]}^{1/2}+\sqrt{\frac{8}{m_{0}}E_{\pi^{⊗N}}[∥\nabla U^{⊗N}\left(x\right){{∥}^{2}]}^{1/2}}. \end{array}$$

Finally, if we increase m(α,N), λ(α,N) increases. Thus, since m(α,N)≥m(α=0,N), we obtain λ(α,N)≥λ(α=0,N).

### Appendix M.2. Computational Complexity

To derive the computational complexity, for simplicity, we assume that δ≤h and We also set $\alpha^{2}\leq h$ for simplicity. This means that the variance of the stochastic gradient is small enough and we use small α. Then the bias is

(A185) $$\begin{array}{cc} \left|E\frac{1}{N}\sum_{n=1}^{N}f\left(X_{k}^{\left(n\right)}\right)-\int_{R^{d}}fd\pi \right| & \leq L_{f}\sqrt{\frac{2}{\lambda (\alpha ,N)}}\sqrt{e^{-\lambda (\alpha ,N)kh}\mathrm{KL}\left(\mu_{0}|\pi \right)+\frac{C_{3}\left(\alpha \right)}{\lambda (\alpha ,N)}}\\ \; & \leq L_{f}\sqrt{\frac{2}{\lambda (\alpha ,N)}}\left(\sqrt{e^{-\lambda (\alpha ,N)kh}\mathrm{KL}\left(\mu_{0}|\pi \right)}+\sqrt{\frac{C_{3}\left(\alpha \right)}{\lambda (\alpha ,N)}}\right), \end{array}$$

where

(A186) $$\begin{array}{cc} \; & C_{3}\left(\alpha \right):=h\left(2\frac{dM}{\beta }\left(m+4M\right)+8C_{0}^{\prime }\left({\left(1+h^{1/2}\right)}^{2}+1\right)\right), \end{array}$$

(A187) $$\begin{array}{cc} \; & C_{0}^{\prime }:=2\left(M^{2}\left(\kappa_{0}+2\left(1\vee \frac{1}{m}\right)\left(b+2{\left(1+h^{1/2}\right)}^{2}B^{2}+\frac{d}{\beta }\right)\right)+B^{2}\right). \end{array}$$

Then we define

(A188) $$\begin{array}{c} C_{3}^{\prime }:=2\frac{dM}{\beta }\left(m+4M\right)+8C_{0}^{\prime }\left({\left(1+h^{1/2}\right)}^{2}+1\right), \end{array}$$

and use the step size that satisfies $h=\frac{\lambda (\alpha ,N)\xi }{2\sqrt{2}C_{3}^{\prime }L_{f}}$. Then when we use

(A189) $$\begin{array}{c} k\geq \frac{2}{\lambda (\alpha ,N)h}\ln\frac{L_{f}}{\xi }\sqrt{\frac{\mathrm{KL}(\mu_{0}|\pi )}{2\lambda (\alpha ,N)}}, \end{array}$$

we have

(A190) $$\begin{array}{cc} \left|E\frac{1}{N}\sum_{n=1}^{N}f\left(X_{k}^{\left(n\right)}\right)-\int_{R^{d}}fd\pi \right| & \leq \frac{\xi }{2}+\frac{\xi }{2}\leq \xi . \end{array}$$
